# A Phloem-Feeding Insect Transfers Bacterial Endophytic Communities between Grapevine Plants

**DOI:** 10.3389/fmicb.2017.00834

**Published:** 2017-05-15

**Authors:** Sebastiàn Lòpez-Fernàndez, Valerio Mazzoni, Federico Pedrazzoli, Ilaria Pertot, Andrea Campisano

**Affiliations:** ^1^Research and Innovation Centre, Fondazione Edmund MachSan Michele all'Adige, Italy; ^2^Infection Biology Department, Institute of Microbiology, Technische Universität BraunschweigBraunschweig, Germany; ^3^Department Microbial Drugs, Helmholtz Centre for Infection ResearchBraunschweig, Germany; ^4^Technology Transfer Centre, Fondazione Edmund MachSan Michele all'Adige, Italy; ^5^Center Agriculture Food Environment, University of TrentoTrento, Italy

**Keywords:** endophytes, pyrosequencing, molecular ecology, insects, grapevine

## Abstract

Bacterial endophytes colonize the inner tissues of host plants through the roots or through discontinuities on the plant surface, including wounds and stomata. Little is known regarding a possible role of insects in acquiring and transmitting non-phytopathogenic microorganisms from plant to plant, especially those endophytes that are beneficial symbionts providing plant protection properties and homeostatic stability to the host. To understand the ecological role of insects in the transmission of endophytic bacteria, we used freshly hatched nymphs of the American sap-feeding leafhopper *Scaphoideus titanus* (vector) to transfer microorganisms across grapevine plants. After contact with the vector, sink plants were colonized by a complex endophytic community dominated by Proteobacteria, highly similar to that present in source plants. A similar bacterial community, but with a higher ratio of Firmicutes, was found on *S. titanus*. Insects feeding only on sink plants transferred an entirely different bacterial community dominated by Actinobacteria, where *Mycobacterium* sp., played a major role. Despite the fact that insects dwelled mostly on plant stems, the bacterial communities in plant roots resembled more closely those inside and on insects, when compared to those of above-ground plant organs. We prove here the potential of insect vectors to transfer entire endophytic bacterial communities between plants. We also describe the role of plants and bacterial endophytes in establishing microbial communities in plant-feeding insects.

## Introduction

Plants are open systems that constantly acquire water and nutrients from the soil and interact with the vast biological diversity of the surroundings (Médiène et al., 2011). This diversity encompasses other plants, animals (i.e., protozoa, annelids, nematodes, arthropods, and vertebrates) and microorganisms. The complex interaction among these diverse players influences crop health and productivity (Atangana et al., 2014). A better understanding of the outcome of these interactions is crucial for improving sustainable crop management and at the same time for identifying new approaches of pest management.

Insects and other invertebrates can transmit diverse microbial plant pathogens (e.g., viruses, phytoplasmas, fungi, and bacteria). Most insect vectors belong to the Hemiptera, an order characterized by piercing and sucking mouthparts that enable feeding from phloem or xylem vessels and, consequently, allow them to acquire and transmit phytopathogens. For example planthoppers and leafhoppers can transmit numerous phytoplasmas, viruses and bacteria (Harris and Maramorosch, 1980). The transmission of insect-borne pathogens and the ecological role of insects as vectors of pathogenic microorganisms have been deeply studied in numerous crops (Weintraub and Beanland, 2006). Mechanistically, there are similarities (modes of acquisition and delivery) in the insect-mediated transmission of individual mutualists and pathogens between plants (Bright and Bulgheresi, 2010; Pèrez-Brocal et al., 2013). However, little is known about the effects of transmitting entire communities of mutualist symbionts and the implications of this transmission in plant host fitness. In addition, information regarding the potential use of transmitted mutualists as a prophylactic tool in plant protection and the ecological implications of a possible natural inoculation with such microorganisms by phloem-feeding insects is lacking.

We chose the American grapevine leafhopper, *Scaphoideus titanus* (Hemiptera: Cicadellidae), as insect model because this species has been largely studied as vector of the flavescence dorée phytoplasma (FDP). *S. titanus* is monovoltine and specialist on grapevine, which means that it lives and feeds on grapevine from hatched nymphs to adults (Chuche and Thiéry, 2014). The life cycle of the insect begins in summer with the egg laying in the bark of woody stems of grapevine, followed by a winter diapause with gradual hatchings occurring from May to early August. Nymphs (five instars) remain most of the time on the abaxial side of leaves of the plant they hatched on. Under laboratory conditions, at a temperature of 23–25°C, the time lapse from egg hatching to adulthood is ~30 days. The adults can live for several weeks and females survive on average 60 days (Jermini et al., 2015). *S. titanus* is mainly a phloem feeder, although mouth stylets can evenly pierce both phloem and xylem vessels (Chuche et al., 2011). While feeding, the insect can acquire FDP that can be then transmitted to other grapevines in a persistent-propagative manner (Foissac and Wilson, 2009). The transmission process includes an incubation period of about 1 month during which phytoplasmas multiply, mostly in the fore- and mid-gut, and accumulate in the salivary glands until they reach a density that permits transmission (Chuche and Thiéry, 2014). The efficiency of FDP acquisition is correlated with phytoplasma titer in the source plant (Galetto et al., 2016). The transmission is non-transovarial, which means that newborn nymphs do not carry the microorganism, but rather they acquire it from infected plants. *S. titanus* engages in multiple symbioses with bacteria, including *Cardinium* sp., *Asaia* sp., and yeast-like endosymbionts (Sacchi et al., 2008). Endophytes asymptomatically colonize the inner tissues of plants (Schulz and Boyle, 2006). Plant colonization mechanisms of bacterial endophytes are complex and symbiosis genes in the genomes of the microbe, inter-kingdom signaling between the plant and the bacterium and plant immunity may play important roles in it, as is the case in many other plant-microorganisms interactions (Iniguez et al., 2005; Reinhold-Hurek and Hurek, 2011; Kusari et al., 2015). The colonization of the plant may result in effects that span from plant growth promotion by nitrogen fixation (Santoyo et al., 2016) to antagonistic properties against plant pathogens (Rabha et al., 2014) and synthesis of exogenous plant hormones that mediate developmental processes in the plant (Khan et al., 2012). Colonization of bacterial endophytes is tissue-specific (Quadt-Hallmann et al., 1997). While many endophytic bacteria can infect and colonize the plant tissues through the roots and move up to the stems (Compant et al., 2008, 2013), some endophytes are known to penetrate the leaves of the plant, possibly through stomata (Compant et al., 2010). In addition, vertical transmission of endophytes has also been demonstrated by the fact that colonized seeds can be a major source of the plant's endomicrobiome (Truyens et al., 2015).

The use of endophytes for disease biocontrol has been postulated in diverse symbiosystems and the effectiveness of endophytes for plant protection and plant growth promotion has been demonstrated (Mercado-Blanco and Lugtenberg, 2014). However, the transmission to plants of beneficial bacteria by insects is still poorly understood. Evidence suggests the transmission of endosymbionts of *S. titanus* (namely *Asaia* sp. and *Cardinium* sp.) through feeding (Gonella et al., 2015). These microorganisms can also be transferred from insect to insect by the venereal route, during copulation and then from insect to plant by feeding. Whether or not these symbionts can survive as endophytes of plants is still unclear. In addition, reports show the horizontal transmission of a common bacterial endophyte, *Methylobacterium mesophilicum*, to *Catharanthus roseus* plants through the leafhopper *Bucephalogonia xanthophis* (Gai et al., 2009). In this work, the bacterium isolated as an endophyte from citrus plants was transformed with an enhanced Green Fluorescent Protein (eGFP)-encoding plasmid, and then transference experiments were set up where the bacterium was tracked with the eGFP signal inside the plants and in the insect.

The transmission of endophytes by insects is a promising subject of study, not only because it may allow the reconstruction of an important step in their ecology, but also because it may enable the efficient delivery of beneficial microorganisms to crops. For this reason, the aim of this work was to assess the transmission by *S. titanus* of the endophytic bacterial community from grapevine plants naturally colonized by endophytes to micropropagated, bacteria-free grapevine plantlets. Using 454 sequencing and qPCR assisted tracking of endophytes, the structure of the endophytic bacterial community was elucidated. In addition, the role of *S. titanus* as vector of bacterial endophytic communities was established. Moreover, the effect of plant and insect hosts in endophytic community structure revealed interactions in the tri-partite system (source plant, sink plant and insect vector).

## Materials and methods

### Plant material

Four 2-year-old grapevine (*Vitis vinifera* L.) plants (cv. Pinot noir grafted on Kober 5BB) were grown under greenhouse-controlled conditions at 24 ± 1°C, 70 ± 10% relative humidity (RH) and a photoperiod of 16L:8D h. Plants were grown in pots on an organic plant substrate and were not treated with any pesticides for the entire course of the experiments. These plants are hereafter referred to as “source” (SRC), since they host the typical complex microbial community of plants grown under natural conditions (Campisano et al., 2014a).

A total of 35 *in vitro* axenically micropropagated grapevine plantlets cv Pinot noir clone I-SMA 185 Cover—[Associazione costitutori viticoli italiani (ACOVIT); Coveri, 1992] were prepared. Homogeneous (mean weight = 0.409 g; standard deviation = 0.058 g) and coeval healthy plantlets with at least three leaves were selected for the experiments. Briefly, the plantlets were micropropagated in cylindrical glass tubes on complete Murashige-Skoog (MS) medium pH 5.6 supplemented with 3% sucrose and 0.6% microagar (Duchefa biochemie, The Netherlands). Explants with one node and internode were incubated in a growth chamber for 51 days at 21 ± 1°C, 16L:8D h photoperiod and a photon irradiance of 50 μm s/m^2^. These *in vitro* plantlets are hereafter referred to as “sink” plants (SNK) and they represent the plants where the bacterial community will be delivered. To further exclude any bacterial presence in the tissues, 10 of these 35 SNK were used as controls.

### Insects

*S. titanus* eggs originated from 2-year-old grapevine canes collected from organic farms in Northern Italy (Villazzano, Trento, Italy, 46°05′N, 11°14′E) during the first week of December 2014 and stored in a cool chamber (4 ± 1°C). Starting from the beginning of April 2015, bundles of canes (0.5 kg) were weekly placed inside plastic boxes containing humid Perlite (Perlitech, Italy) in a climate chamber (24 ± 1°C, 16L:8D h photoperiod, 75% RH) where, after 30–60 days, eggs gradually hatched. Freshly hatched nymphs (IN) were removed daily and gently transferred to a SRC using a suction aspirator.

### Experimental design

**Figure 1:**
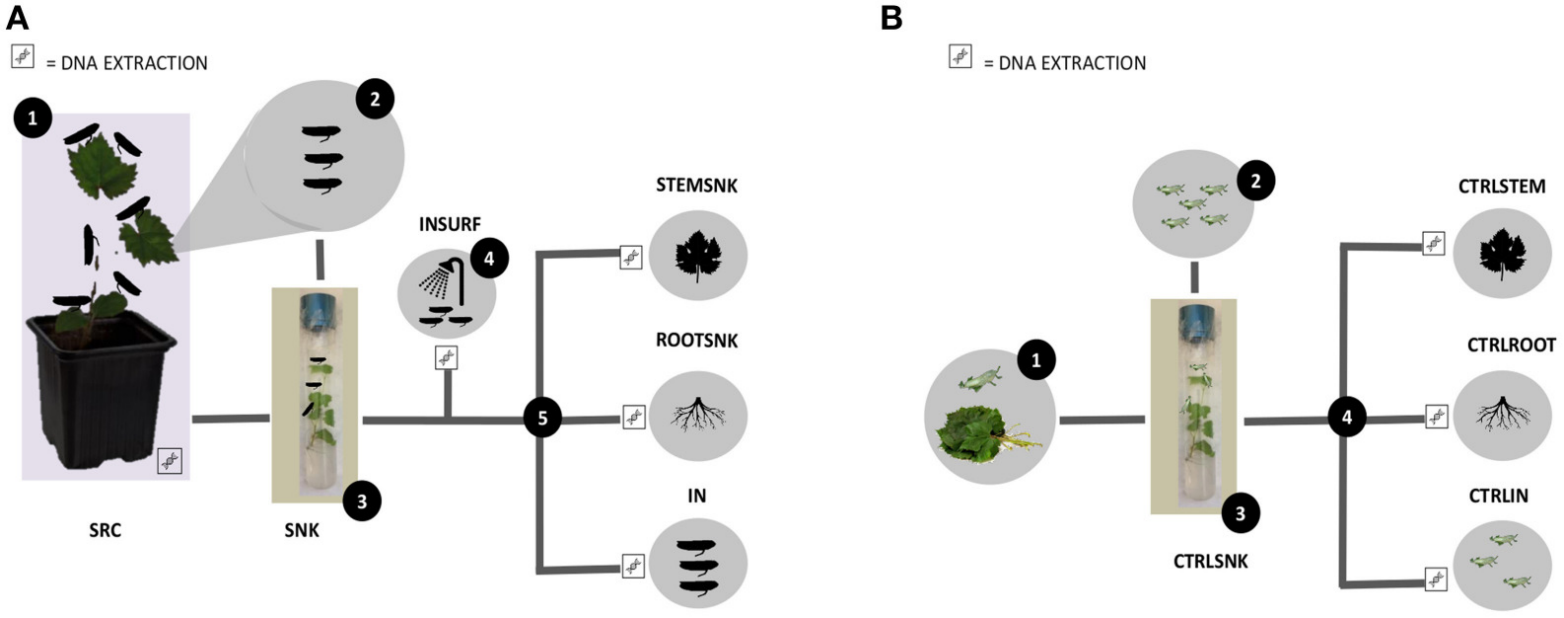
**Experimental design. (A)** Set up of endophyte transmission experiments through *Scaphoideus titanus*—Tests. (1) Source plants (SRC) were infested with insects (IN); (2) insects were placed on sink plants (SNK); (3) insects and sink plants were incubated; (4) surfaces of insects were washed (INSRUF); (5) insects, roots (ROOTSNK), and stems (STEMSNK) of sink plants were separated. Surface sterilization was performed before each DNA extraction step. **(B)** Set up of endophyte transmission experiments through *S. titanus*—Controls. (1) Control insects (CTRLIN) were left to hatch on grapevine trunks; (2) control insects were placed in control sink plants (CTRLSNK); (3) control insects and control sink plants were incubated, (4) control insects, control roots (CTRLROOT), and control stems (CTRLSTEM) were separated. Surface sterilization was performed before each DNA extraction step.

The transmission experiment (Figure 1) was carried out independently four times, using new plants and insects. Four SRC were kept under constant environmental conditions as mentioned above. Then, 96 IN were placed and confined onto four well developed SRC leaves, where restricted areas were delimited by small cages (four cages per plant, with six INs each for a total of 24 insects per plant) made out of a mesh sleeve (250 μm mesh size) and a supporting plastic cylindrical structure (ø = 10 cm; h = 20 cm). The IN were let feed and grow for 14 days until they reached a stage between the third and fourth nymphal instar. Then, out of the 96 IN previously transferred, 48 individuals were collected from the SRC (12 IN per SRC) and transferred to 16 SNK (four SNK with three IN per each SRC). In addition, an insect-free SNK per each SRC was included as a sterility control of the replicate. Before transferring the IN, surface of the MS medium supporting the micropropagated SNK was overlaid with 1 ml of sterile melted paraffin (Sigma-Aldrich, Germany) in order to prevent the contamination of the growth medium and the roots by microorganisms carried by IN. In this way, we could assure that no contact between the roots or the growth medium was taking place. Once transferred to the SNK, IN were allowed to feed for 10 days at 21°C, 16L:8D h photoperiod and a photon irradiance of 50 μm s/m^2^ until they were fifth instar nymphs or adults.

As control, five SNK (CTRLSNK) were each infested with five freshly hatched nymphs (CTRLIN) that had not been previously reared on SRC, but were feeding only on SNK (Figure 1B). In addition, from the remaining ten SNK, five were used to probe for bacterial DNA in the plant's tissues. Total plant DNA was extracted using the method previously described (Campisano et al., 2014a) and the extracted DNA was amplified using the primer pair 799F/1520R (Yousaf et al., 2014). Since the five tested plants were PCR negative (no amplification of bacterial 16SrDNA gene), they were considered bacteria-free. Although primers 799F and 1520R are a universal pair for 16SrDNA amplification, still some prokaryotes might not have been detected. Thus, the last five SNK plantlets were used to control microbial contamination inside the tissues. SNK were incubated under the same conditions without IN. Then, plants were crushed in a sterile mortar with 1 ml phosphate buffer saline 1X, pH 7.2, and the resulting extract was plated on Luria-Bertani agar (LBA; Sigma Aldrich, Germany) and incubated at 30°C for 5 days, after which no growth was recorded.

After the incubation period, all SNK and IN were aseptically removed from the glass tubes. SNK were cut into stems (STEMSNK) and roots (ROOTSNK); CTRLSNK were likewise cut into CTRLROOT and CTRLSTEM samples. IN were washed with distilled sterile water by thoroughly vortexing in order to dislodge the majority of surface-adhering bacteria. The bacterial cells in the washing water (INSURF) were pelleted by centrifugation at 13,000 rpm on a tabletop centrifuge and stored at −20°C before extracting the DNA. All SRC, IN, CTRLIN, STEMSNK, ROOTSNK, CTRLSTEM, and CTRLROOT were then surface-sterilized by successive washing in 98% ethanol for two min, 4% sodium hypochlorite for 2 min and 70% ethanol for 2 min as described previously (Pancher et al., 2012), and then rinsed three times with distilled sterile water. The water from the final washing step of all samples was plated on LBA and incubated for 5 days at 30°C to check for microbial growth as a proxy for surface disinfection efficacy.

### DNA extraction, 16SrDNA amplification, and pyrosequencing

After sterilization, SRC, IN, CTRLIN, STEMSNK, ROOTSNK, CTRLSTEM, and CTRLROOT were aseptically transferred to sterile stainless steel capsules containing steel beads. The material was frozen in liquid nitrogen for 5 min and crushed in a Retsch MM200 tissue lyser (Qiagen, The Netherlands) for 2 min at a frequency of 25 hertz. The resulting powder was weighted and then deoxyribonucleic acids were extracted using the FastDNA™ SPIN Kit for Soil (MP, United States) according to manufacturer's instructions. DNA from the INSURF samples was extracted with the same kit after pelleting and suspending the cells in extraction buffer before workup.

DNA was then quantified in an UV-VIS nanodrop 8,000 spectrophotometer (Thermo Fischer Scientific, United States) and PCR-amplified using the primer pair 799F (AACMGGATTAGATACCCK) and 1520R (AAGGAGGTGATCCAGCCGCA) targeting the V5–V9 16S rDNA hypervariable regions without amplification of plastid DNA. These primers bear 454 adaptors and a sample-specific barcode on the forward primer. PCR was performed using the Roche high fidelity Fast Start PCR system (Roche, Switzerland) in a final volume of 25 μl. The following volumes, reagents and concentrations were used: 2.5 μl amplification buffer 10X, 5 μl MgCl_2_ 25 mM, 0.5 μl reverse primer 10 μM, 0.5 μl forward primer 10 μM, 2.5 μl dNTPs 25 mM, 1 μl DMSO, 2.5 Bovine serum albumin (BSA) 10 mg/ml, 0.4 μl HI-FI Taq polymerase 5 U/μl and water. DNA was adjusted to an initial concentration of 3 ng/μl and for some samples dilutions of 1:10 were used in order to obtain optimal amplification. Thirty cycles of PCR were carried out according to the manufacturer's instructions with conditions for amplification as follows: 5 min of initial denaturation at 95°C, 30 s at 95°C, 1 min for annealing at 53°C, 2 min for extension at 72°C, and a final extension step 10 min at 72°C. PCR products were separated in a 1.5% agarose gel stained with SYBR® Safe DNA Gel Stain (Thermo Fisher Scientific, United States), and visualized on a Gel Doc XR+ system (BiO-RAD, United States). The appropriate amplification bands were excised from the gel. DNA was recovered using the PureLink Quick gel extraction Kit (Thermo Fisher Scientific, United States) according to manufacturer's instructions. Three different amplifications for each sample were performed and the PCR products were purified from gel and pooled together for pyrosequencing. Amplicons were quantified with quantitative PCR using the library quantification kit Roche 454 Titanium (KAPA Biosystems, United States) and pooled in equimolar ratio in the final amplicon library. Pyrosequencing was carried out on the Roche GS FLX+ system using the new XL+ chemistry dedicated to long reads of up to 800 bp, following the manufacturer's recommendations.

### Bacterial 16SrDNA amplicon demultiplexing and statistical analysis

Outputs from the 454 pyrosequencing were analyzed using the “Quantitative Insights into Microbial Ecology (QIIME)” pipeline, version 1.9.0 (Caporaso et al., 2010b). The analysis consisted of decoding the sequence flowgram files (SFF) and producing fasta and quality files with which length of sequences and quality of reads were checked. Amplicon sequences were demultiplexed (assigned to sample pools) according to their barcoded primer. Only bacterial sequences at least 300 nt long were retained. Sequences were truncated when the quality score in a 50 nt long sliding window went below 25.

Chimeric PCR products were identified using USEARCH 6.1.544 (Edgar, 2010). Operational taxonomic units (OTUs) were picked using a threshold identity of 97% and the Greengenes database, August 2013 version (DeSantis et al., 2006). USEARCH cluster seeds were used as representatives for OTUs, while taxonomy was assigned using USEARCH and the Greengenes database as a template. Sequences assigned to chloroplasts and mitochondria were removed. OTUs represented by only one or two reads (singletons and doubletons) were removed from the OTU tables. The amplicons were then aligned *de novo* using pynast (Caporaso et al., 2010a) and the alignment was used to generate a phylogenetic tree.

From pyrosequencing we obtained 1,404,963 reads from the whole set of samples, with a median of 13,271 reads per sample. After the first quality control steps where we removed short sequences (<200 nt), mis-sequenced fragments and mutated amplicons, only 1,024,657 sequences were left. Following removal of chimeric sequences using the Usearch algorithm, 871,497 remained as non-chimeric sequences. Here, a maximum of 31,039 sequences for IN samples and a minimum of 57 sequences for CTRLIN samples were obtained, and a mean of 12,939 sequences for all the samples.

For clustering OTUs, we picked a representative set of sequences that further represented the OTU with 97% accuracy, resulting in 2,005 grouped sequences available for analysis. Some of the sequences obtained were found to be of plant nature (plastid sequences) and were removed, leading to a final count of 1,923 sequences. From those, we removed the sequences that were represented in <1% of the total population, obtaining an OTU table with a total of 447 OTUs that were defined as clusters composed of three or more sequences.

Alpha- and beta-diversity were estimated on multiple OTU tables rarefied to 1,300 reads (considering the sample with the lowest number of reads). Alpha-diversity differences were tested for statistical significance using 999 Monte Carlo permutations and the *p*-value obtained corrected using the Bonferroni correction for multiple comparisons. Beta-diversity was computed using the phylogenetic unweighted UniFrac distances. PCoA plots rendering sample distances were visualized using Emperor and further drawn in R. A Kruskal–Wallis test was used to assess if the differential distribution of OTUs and taxa was statistically significant for all the variables analyzed. The multivariate test ANOSIM to detect differences between groups of samples was used as implemented in QIIME.

### Transmission and quantification of endophytes through qPCR

To quantify the bacteria transferred by *S. titanus* across plants, we used a similar setting to the one described above. In this case, the source of inoculum is not the SRC, but a bacterial cell suspension of cultivable endophytes isolated from grapevine trunks in a previous work. These bacteria were classified as *Enterobacter ludwigii* EnVs6, *E. ludwigii* EnVs2, and *Pantoea vagans* PaVv9 (Campisano et al., 2015; Lòpez-Fernàndez et al., 2015).

Briefly, these bacterial endophytes were transformed with the eGFP encoding plasmid pMP4655 (Bloemberg et al., 2000) as follows: bacteria were grown on LBA for 48 h at 30°C. Then, 2 ml of super optimal broth amended with sucrose (SOC) were inoculated with a single colony and incubated for 24 h at 30°C and 160 rpm (Hanahan, 1983). Aliquots of 400 μl of this starter culture were inoculated into 40 ml of SOC broth and then incubated for further 24 h at 30°C and 160 rpm. Cells were then centrifuged at 45,895 rpm for 15 min at 4°C, and subsequently suspended in electroporation buffer (glycerol 10%, distilled sterile water maintained at <4°C) for plasmid insertion into the bacterial cells. Three washing steps were performed with electroporation buffer, reducing in halves the resuspension volume. At the end, aliquots of 50 μl of buffered immersed (competent) bacteria were dispensed in tubes and kept at −80°C. Bacterial cells were then gently mixed with 1 μg of the plasmid and incubated on ice for 30 min. Later, the mixture was transferred to 0.2 cm electroporation cuvettes (Biorad, United States) and electroporated at 1,500 mV, 25 μF, and 200 Ω. Cells were immediately immersed in 800 μl of SOC and incubated at 30°C and 160 rpm for 2 h. Cultures were centrifuged and half of the volume discarded. Then, cells were suspended in the remaining volume and plated onto LBA supplemented with tetracycline (20 μg/ml). Transformants were confirmed by amplifying the resistance marker cassette tetA/R, present in the plasmid, with primers directed toward the gene, as previously reported (Møller et al., 2016).

Endophytic cells bearing the pMP4655 were grown on LB for 24 h and cell densities were adjusted to 3 × 10^7^ CFU/ml. Then, cells were cooled down on ice and washed three times with PBS 1X, pH 7.2. After the last washing step, cells were re-suspended in 200 μl of a Tris-EDTA-sucrose pH 8.0 solution (TES: Tris 10 mM, EDTA 1 mM, sucrose 5% w/v) and distributed in the lids of bottomless (replaced by a cotton plug) 1.5 ml plastic tubes (Eppendorf, Germany). Lids were covered with one layer of sterile parafilm (Bemis NA, United States).

*S. titanus* individuals were reared as described above. Insects were transferred to plastic tubes with the lids hanging upside down, and left to feed on the eGFP-tagged bacteria for 5 days. In this setting, insects punched the parafilm layer on the lid, releasing and feeding from the bacteria-rich TES solution.

After feeding, insects were transferred to *in vitro* micropropagated grapevine plantlets, as described in the experiments above. Per each bacterium tested we performed one set of four replicates consisting of four plants infested with three insects per plant plus one negative control where no insects were transferred. Replicates were incubated for 5 days. At the end of the incubation, the insects, roots and stems were collected separately. DNA from the insects and plants was extracted using the NucleoSpin® Plant II kit (for ROOTNSK, STEMSNK, CTRLROOT, and CTRLSTEM) and the Nucleospin® Tissue (for IN and CTRLIN) according to manufacturer instructions (Macherey-Nagel, Germany). DNA was quantified as described above.

Bacterial DNA, including the pMP4655 eGFP encoding plasmid, was quantified on a Roche LightCycler® 480 Real-Time PCR (Roche, Switzerland) with the platinum SYBR Green qPCR superMix-UDG (Thermo Fisher Scientific, United States). The following amplification protocol was used: 1 hold at 50°C activation (UDG incubation) for 5 min, 1 hold at 95°C for 5 min activation, 40 cycles at 95°C for 30 s for melting and at 58°C for 45 s for annealing and extension. An analysis of melting curves at 95°C for 5 s, followed by a down until 55°C for 1 min, was performed to check for specificity of the reaction. Absolute quantification of eGFP gene copies in plants and insects was done based on interpolation from a standard curve obtained with serial 10-fold dilutions of the eGFP gene (from 3 × 10^6^ to 3 × 10^1^ eGFP gene copies/μl) in DNA of control plants or insects, respectively.

### Nucleotide sequence accession numbers

The sequencing output is deposited at the European Nucleotide Archive (ENA at https://www.ebi.ac.uk), and can be found under the accession number ERS1629270, study name PRJEB20051.

## Results

Our data show that the whole microbial communities living on the plant are transported between plants by insect vectors. By feeding and touching the plant, insects acquire a set of microorganisms that radically differs from those they have at hatching. Insects are able to carry and transfer this set to other plants they dwell and feed upon.

### Structure of the community in the tested grapevine symbiosystem

**Figure 2:**
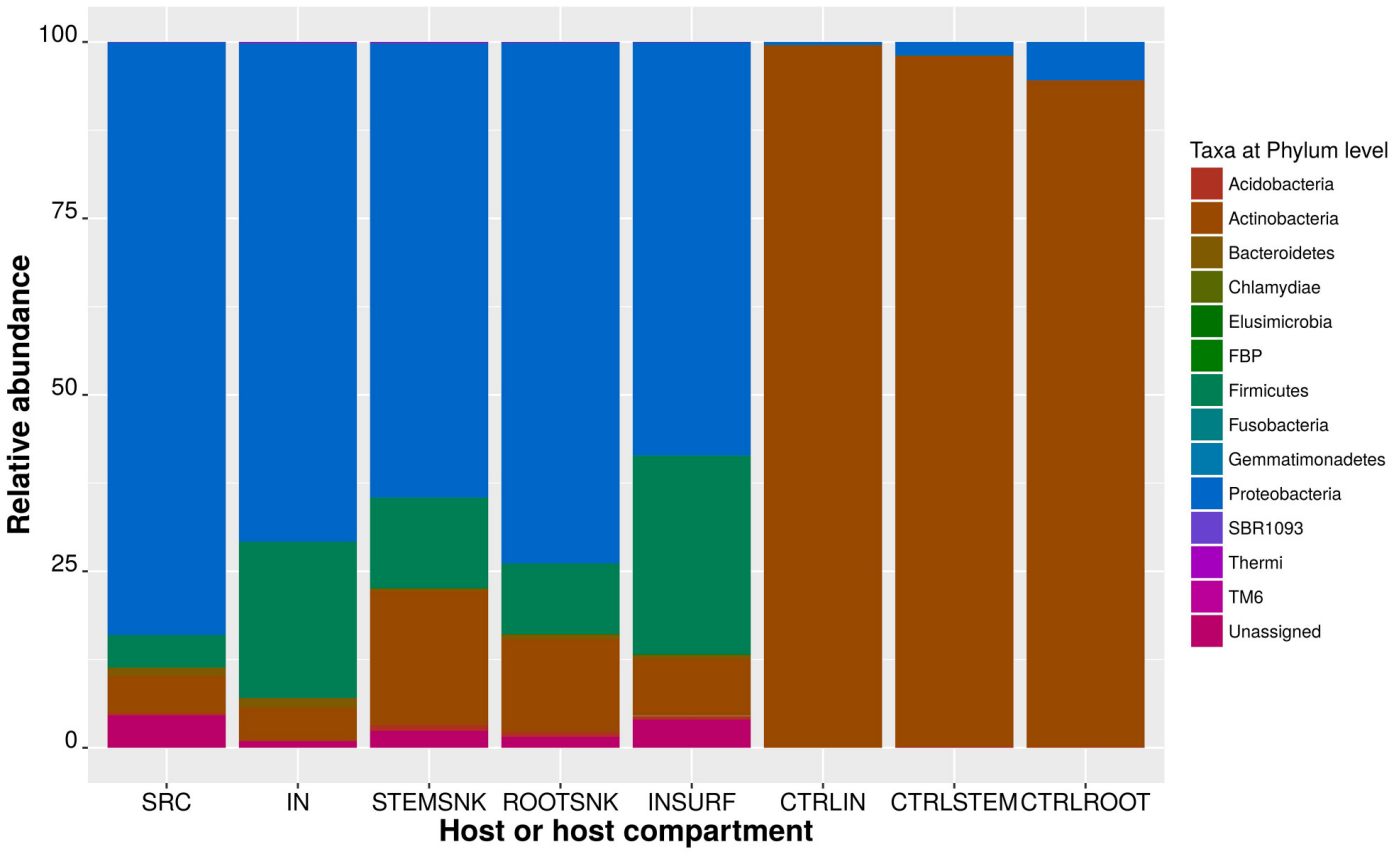
**Relative abundance of OTUs assigned at the phylum level**. Stacked bar plots represent the percentages of 216 members of the OTU table in biom format. In the legend, unassigned correspond to OTUs whose taxonomy could not be clarified at the 97% confidence using the Greengenes database.

The relative abundances of bacterial phyla varied between hosts. The control samples had a distinctively different species composition than the test samples (Figure 2 and Table 1). In the SRC, the bacterial community was mainly composed of Proteobacteria, where the most abundant classes were Beta-, Gamma-, and Alpha-proteobacteria. The rest of the community was composed of Actinobacteria (a majority in the class Actinobacteria), Firmicutes (with most members affiliated with the class Bacilli), Bacteroidetes (the classes Sphingobacteriia and Spirospirae made the majority of the phylum) and, in a smaller proportion, Acidobacteria (represented only by the class Solibacteres), and Chlamydiae (represented only by the class Chlamydiia). Only a small fraction of the OTUs could not be assigned to any particular taxon. The bacterial community of IN was composed mostly of Proteobacteria followed by Firmicutes, Actinobacteria, Bacteroidetes, and Acidobacteria, the latter representing the least abundant phylum. The INSURF community was similar to the inner bacterial microbiota of IN with the majority of OTUs assigned to Proteobacteria, followed by Firmicutes, Actinobacteria, Bacteroidetes, and Acidobacteria. In our analysis, only one OTU was exclusively associated with the INSURF samples. Using Basic Local Alignment Search Tool (BLAST), this sequence was assigned to the Sinobacteraceae, a family that includes the closely related water-spring associated bacterium *Nevskia* sp. This sequence was never detected in any plant sample or inside the insects.

**Table 1:** **Relative abundance of OTUs in the symbiosystem ***S. titanus***—grapevine, assigned at phylum and class level**.

**Phylum**	**Class**	**SRC**	**IN**	**STEMSNK**	**ROOTSNK**	**INSURF**	**CTRLIN**	**CTRLTROOT**	**CTRLSTEM**
Unassigned	Unassigned	4.6	0.9	2.4	1.5	4.0	0.0	0.0	0.1
Acidobacteria	Acidobacteriia	0	0.1	0	0.2	0	0	0	0
	DA052 clade	0.3	0.1	0.8	0.5	0.4	0	0	0
	Solibacteres	0	0	0	0	0	0	0	0
	Total	0.3	0.2	0.8	0.7	0.4	0.0	0.0	0.0
Actinobacteria	Actinobacteria	5.3	4.6	19	13.1	8.2	99.5	94.5	98
	Thermoleophilia	0	0	0.1	0	0	0	0	0
	Total	5.3	4.6	19.1	13.1	8.2	99.5	94.5	98.0
Bacteroidetes	Bacteroidia	0.1	0	0	0.1	0	0	0	0
	Cytophagia	0	0.1	0.1	0	0	0	0	0
	Flavobacteriia	0	0	0	0	0	0	0	0
	Sphingobacteriia	0.5	0.2	0	0.1	0	0	0	0
	Saprospirae	0.5	1	0.1	0.4	0.4	0	0	0
	Total	1.1	1.3	0.2	0.6	0.5	0.0	0.0	0.0
Chlamydiae	Chlamydiia	0.1	0	0.1	0.1	0.1	0	0	0
	Total	0.1	0.0	0.1	0.1	0.1	0.0	0.0	0.0
Elusimicrobia	Elusimicrobia	0	0	0	0	0.1	0	0	0
	Total	0.0	0.0	0.0	0.0	0.1	0.0	0.0	0.0
FBP	FBP	0	0	0	0	0	0	0	0
	Total	0.0	0.0	0.0	0.0	0.0	0.0	0.0	0.0
Firmicutes	Bacilli	4.5	21.5	12.7	9.7	27.7	0	0	0.1
	Clostridia	0.1	0.6	0.1	0.2	0.4	0	0	0
	Total	4.6	22.1	12.8	10.0	28.1	0.0	0.0	0.1
Fusobacteria	Fusobacteriia	0	0	0	0	0.1	0	0	0
	Total	0.0	0.0	0.0	0.0	0.1	0.0	0.0	0.0
Gemmatimonadetes	Gemmatimonadetes	0	0	0	0	0	0	0	0
	Total	0.0	0.0	0.0	0.0	0.0	0.0	0.0	0.0
Proteobacteria	Alpha	13.9	2.7	5.7	4.4	2.1	0.2	0.2	0.1
	Beta	36.6	29.5	40.9	45.9	31.8	0.2	4.9	0.1
	Delta	0.7	0.3	0.3	0.4	0.5	0	0	0
	Gamma	32.7	38.2	17.3	23	24.1	0	0.3	1.7
	Total	83.9	70.6	64.3	73.8	58.5	0.4	5.4	1.9
SBR1093	VHS-B5-50	0	0	0	0	0	0	0	0
	Total	0.0	0.0	0.0	0.0	0.0	0.0	0.0	0.0
TM6	SJA-4	0	0	0	0.1	0	0	0	0
	Total	0.0	0.0	0.0	0.1	0.0	0.0	0.0	0.0
Thermi	Deinococci	0	0.1	0.1	0	0	0	0	0
	Total	0.0	0.1	0.1	0.0	0.0	0.0	0.0	0.0

Microbiota of the SNK was mostly composed of Proteobacteria, with Beta- and Gamma-proteobacteria being the most abundant classes. Deltaproteobacteria were also present and the rest of the phyla had only few representatives (Table 1). A further analysis of the endophytic community composition in the above- and below-ground compartments revealed differences in the two plant compartments. Proteobacterial OTUs in STEMSNK were highly represented (in order of abundance: Betaproteobacteria, Gammaproteobacteria, Alphaproteobacteria, and Deltaproteobacteria). Actinobacteria, Firmicutes, Acidobacteria, Bacteroidetes, and Chlamydiae were less abundant.

In ROOTSNK, Proteobacteria were also the most abundant OTUs (in order of abundance: Beta-, Gamma-, Alpha-, and Delta-proteobacteria). Actinobacteria, Firmicutes, Acidobacteria, Bacteroidetes, Chlamydia, and the candidate clade TM6 were the least abundant. In contrast, the bacterial community of control plants (CTRLROOT, CTRLSTEM, where freshly hatched insects had fed without prior contact with SRC) was dominated by Actinobacteria, with only a small proportion of Proteobacteria (Beta- and Gamma-, but no Alpha-proteobacteria). The community in CRTLIN was also dominated by Actinobacteria.

### Selected endophytes are transmitted between grapevine plants

Forty OTUs were transferred by IN from SRC to SNK and found both in ROOTSNK and in STEMSNK (Table 2). These were never found in the CTRLROOT or CTRLSTEM, suggesting that they were efficiently transmitted from SRC to SNK by IN. In particular, among the sequences of the main phyla (i.e., Proteobacteria, Actinobacteria, Bacteroidetes, Chlamydiae, and Firmicutes), Proteobacteria were the most highly represented taxon, where the most abundant genera were *Agrobacterium, Paracoccus, Sphingomonas, Erwinia, Pseudomonas, Lysobacter*, and *Stenotrophomonas*.

**Table 2:** **OTUs transmitted from source (SRC) to sink plants (STEMSNK and ROOTSNK) by ***S. titanus*****.

**OTU**	**Phylum**	**Class**	**Order**	**Family**	**Genus**	**Species**
1	Actinobacteria	Actinobacteria	Actinomycetales	Unclassified	Unclassified	Unclassified
2	Actinobacteria	Actinobacteria	Actinomycetales	Corynebacteriaceae	Corynebacterium	durum
3	Actinobacteria	Actinobacteria	Actinomycetales	Geodermatophilaceae	Unclassified	Unclassified
4	Actinobacteria	Actinobacteria	Actinomycetales	Micrococcaceae	Kocuria	palustris
5	Actinobacteria	Actinobacteria	Actinomycetales	Micrococcaceae	Micrococcus	luteus
6	Actinobacteria	Actinobacteria	Actinomycetales	Nocardioidaceae	Unclassified	Unclassified
7	Actinobacteria	Actinobacteria	Actinomycetales	Propionibacteriaceae	Propionibacterium	Unclassified
8	Bacteroidetes	Flavobacteriia	Flavobacteriales	Flavobacteriaceae	Flavobacterium	Unclassified
9	Bacteroidetes	Saprospirae	Saprospirales	Chitinophagaceae	Sediminibacterium	Unclassified
10	Chlamydiae	Chlamydiia	Chlamydiales	Parachlamydiaceae	Unclassified	Unclassified
11	Firmicutes	Bacilli	Bacillales	Bacillaceae	Bacillus	flexus
12	Firmicutes	Bacilli	Bacillales	Staphylococcaceae	Staphylococcus	aureus
13	Firmicutes	Clostridia	Clostridiales	Tissierellaceae	Anaerococcus	Unclassified
14	Proteobacteria	Alphaproteobacteria	Rhizobiales	Phyllobacteriaceae	Unclassified	Unclassified
15	Proteobacteria	Alphaproteobacteria	Rhizobiales	Rhizobiaceae	Unclassified	Unclassified
16	Proteobacteria	Alphaproteobacteria	Rhizobiales	Rhizobiaceae	Agrobacterium	Unclassified
17	Proteobacteria	Alphaproteobacteria	Rhodobacterales	Rhodobacteraceae	Paracoccus	Unclassified
18	Proteobacteria	Alphaproteobacteria	Rhodospirillales	Rhodospirillaceae	Unclassified	Unclassified
19	Proteobacteria	Alphaproteobacteria	Rickettsiales	Unclassified	Unclassified	Unclassified
20	Proteobacteria	Alphaproteobacteria	Sphingomonadales	Sphingomonadaceae	Unclassified	Unclassified
21	Proteobacteria	Alphaproteobacteria	Sphingomonadales	Sphingomonadaceae	Kaistobacter	Unclassified
22	Proteobacteria	Alphaproteobacteria	Sphingomonadales	Sphingomonadaceae	Sphingomonas	Unclassified
23	Proteobacteria	Betaproteobacteria	Burkholderiales	Alcaligenaceae	Achromobacter	Unclassified
24	Proteobacteria	Betaproteobacteria	Neisseriales	Neisseriaceae	Unclassified	Unclassified
25	Proteobacteria	Betaproteobacteria	Neisseriales	Neisseriaceae	Unclassified	Unclassified
26	Proteobacteria	Betaproteobacteria	Neisseriales	Neisseriaceae	Kingella	Unclassified
27	Proteobacteria	Betaproteobacteria	Neisseriales	Neisseriaceae	Neisseria	Unclassified
28	Proteobacteria	Betaproteobacteria	Neisseriales	Neisseriaceae	Neisseria	cinerea
29	Proteobacteria	Deltaproteobacteria	Unclassified	Unclassified	Unclassified	Unclassified
30	Proteobacteria	Deltaproteobacteria	Myxococcales	0319-6G20	Unclassified	Unclassified
31	Proteobacteria	Gammaproteobacteria	Alteromonadales	Alteromonadaceae	Marinobacter	Unclassified
32	Proteobacteria	Gammaproteobacteria	Enterobacteriales	Enterobacteriaceae	Unclassified	Unclassified
33	Proteobacteria	Gammaproteobacteria	Enterobacteriales	Enterobacteriaceae	Erwinia	Unclassified
34	Proteobacteria	Gammaproteobacteria	Legionellales	Unclassified	Unclassified	Unclassified
35	Proteobacteria	Gammaproteobacteria	Pseudomonadales	Pseudomonadaceae	Pseudomonas	nitroreducens
36	Proteobacteria	Gammaproteobacteria	Xanthomonadales	Sinobacteraceae	Unclassified	Unclassified
37	Proteobacteria	Gammaproteobacteria	Xanthomonadales	Xanthomonadaceae	Luteimonas	Unclassified
38	Proteobacteria	Gammaproteobacteria	Xanthomonadales	Xanthomonadaceae	Lysobacter	Unclassified
39	Proteobacteria	Gammaproteobacteria	Xanthomonadales	Xanthomonadaceae	Stenotrophomonas	Unclassified
40	TM6	SJA-4	Unclassified	Unclassified	Unclassified	Unclassified

In contrast, the most abundant phyla in CTRLIN were Actinobacteria, especially the genera *Mycobacterium, Gordonia, Nocardia, Rhodococcus*, and *Williamsia*. CTRLROOT and CTRLSTEM hosted a community that resembled that of the CTRLIN (Table 1). For example, the genus *Nocardia* was detected in all sample types, but its prevalence was lowest in CTRLROOT samples. Likewise, *Rhodococcus* and *Aeromicrobium* were less abundant in CTRLROOT and CTRLSTEM than in CTRLIN. An exception was the genus *Williamsia*, which was more abundant in CTRLROOT and CTRLSTEM than in CTRLIN.

### Endophytic communities move from stems to roots after the transfer process

To identify colonization dynamics of transmitted endophytes, we compared the community in above- and below-ground parts of the plant (ROOTSNK vs. STEMSNK). In our experimental setup, plant roots were externally separated from the stems by a paraffin layer placed to avoid surface contamination of the synthetic medium. Our data thus indicate that several OTUs were detected both in STEMSNK and ROOTSNK, including *Streptococcus, Steroidobacter, Ralstonia, Pseudomonas*, and *Methylobacterium*. ROOTSNK had more Proteobacteria than STEMSNK, while Actinobacteria were more abundant in STEMSNK than in ROOTSNK.

STEMSNK samples were dominated by Proteobacteria (Beta- Gamma-, Alpha-, and Delta-proteobacteria). Actinobacteria, Firmicutes, Acidobacteria, Bacteroidetes, and Chlamydiae were the least abundant phyla. Similarly, in ROOTSNK Proteobacteria represented most of the community, followed by the Actinobacteria, Firmicutes, Acidobacteria, Bacteroidetes, Chlamydiae, and the candidate phylum TM6.

To verify for insect transmission, we also evaluated the endophytic communities in above- and below-ground parts of control sink plants (CTRLSTEM and CTRLROOT) in contact only with CTRLIN. The bacterial community of CTRLSTEM and CTRLTROOT, where freshly hatched CTRLIN had fed without prior contact with SRC, was dominated by Actinobacteria with a small proportion of Proteobacteria (Beta- and Gamma-, but not Alphaproteobacteria were present).

### Insects change the community structure during passage from source to sink plants

To identify shifts in bacterial community composition in the transferring process, we analyzed diversity for every host type and compared their significance at a large scale (higher taxonomic hierarchy or phylum level) and in some cases at a small scale (genus level).

**Figure 3:**
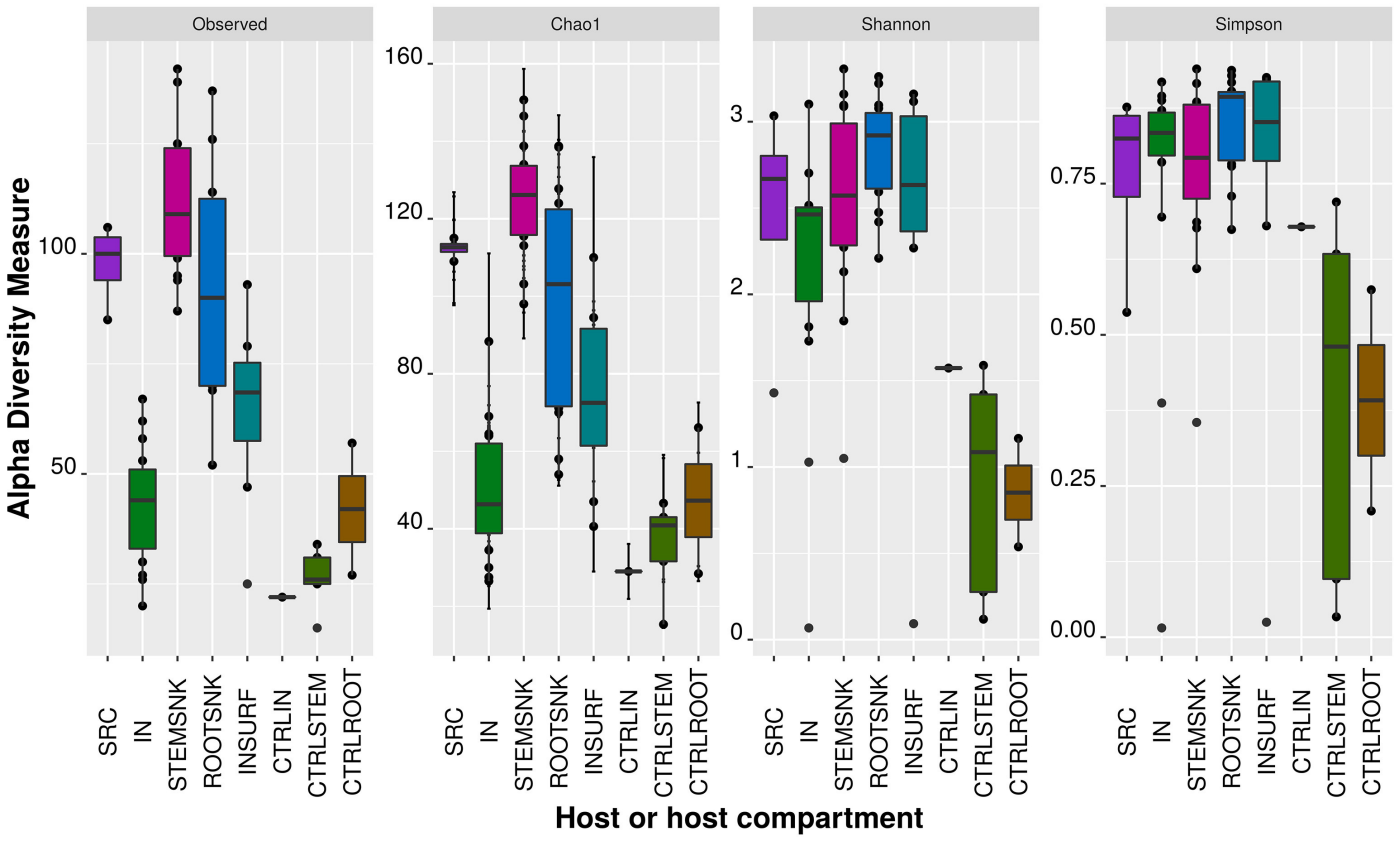
**Alpha diversity measures on transmission experiments**. Alpha diversity indexes (Richness: Observed species, *Chao1*; Diversity: Shannon-Wiener, and Simpson) were calculated on the OTU table at the phylum level. Reads were rarefied to 1,300 sequences to have an even representation of OTUs in each sample category (or host).

The largest diversity was present in ROOTSNK followed by STEMSNK. The third most diverse microbiota was that of SRC followed by IN (Figure 3). In terms of richness, a large number of new species was detected in SRC (observed species and *Chao 1* index), though the number of species in IN acquired during feeding was less than a half of what it had been in the SRC (Figure 3 and Supplementary Figure 1). In addition, variance within samples was larger in IN as compared to SRC, suggesting differences in species composition in each sample. In STEMSNK the richness increased considerably more than in the below-ground part of the plant, with a high variance within samples, suggesting that the community that was previously stable in SRC was disturbed in the SNK after acquisition and transmission by IN. When looking at Shannon-Wiener and Simpson's indexes, diversity was found to be higher in SRC (abundance of new species is larger and even) and lower in IN (Supplementary Table 1A). When the community was transferred to STEMSNK, its diversity increased. The standard variation within samples increased from SRC (0.878) to IN (10.52), suggesting less evenness per sample. When passing to the SNK, the standard variation decreased (STEMSNK = 10.52 and ROOTSNK = 0.430), pointing to a recovery of the community when moving from the insect to the plant host. Although INSURF had few new species per sample, the diversity was near to that of the other samples, hinting at the possibility of an input of insect surface-associated bacteria to the endophytic community (Supplementary Table 1A). The diversity of the controls differed from that of the treated samples. The lowest diversity and richness were recorded for CTRLIN, CTRLROOT, and CTRLSTEM. Rarefaction curve analysis (Supplementary Figure 1) confirmed that the richest samples were SRC and the compartments of the sink plants (STEMSNK and ROOTSNK), followed by the INSURF microbiome and by the inner microbiome IN. CTRLROOT were the poorest samples in terms of new species because no new OTUs being discovered after a sampling effort of 1,505 sequences/sampling. The rarefaction analysis showed that a large proportion of the endophytic bacterial community was represented in our analysis, since all curves showed a *plateau* starting at and saturated after the threshold of 1,505 sequences/sampling.

Statistical analysis using non-parametric *t*-tests on richness and diversity indexes unveiled interesting patterns that delineated the transmission pathway. With the *Chao1* richness estimator, we found significant differences between SRC and IN, STEMSNK and CTRLSTEM, IN and ROOTSNK, STEMSNK and IN, INSURF and CRTLSTEM, and CTRLSTEM and ROOTSNK (Supplementary Table 1B).

Statistical comparisons of the Shannon-Wiener indexes per sample group showed significant differences between CTRLSTEM and STEMSNK as well as differences between the CTRLSTEM and the ROOTSNK. This further confirmed the host effect on the bacterial community. As we expected, IN and ROOTSNK showed a significant diversity shift, suggesting that during the passage from insect to sink plants the community was shaped in a host-dependent manner. A tendency to differential diversities was detected between CTRLSTEM and ROOTSNK, although this difference was borderline (Supplementary Table 1B).

The non-parametric *t*-test analysis showed that the community of INSURF and IN was mainly composed of the same taxa, with additional presence of particular groups, namely Fusobacteria and Gemmatimonadetes, on the surface of the insect. Although both sample types had overall the same taxa composition, their abundances also varied. The taxa that differed in their abundances were *Actinomyces, Burkholderia, Haemophilus parainfluenzae, Kocuria palustris, Streptococcus* sp., Neisseraceae, *Kingella* sp. and a member of the Micrococcacea, whose taxonomy remains to be determined (Supplementary Table 1).

### Endophytic community composition shifts in a host-specific manner

**Figure 4:**
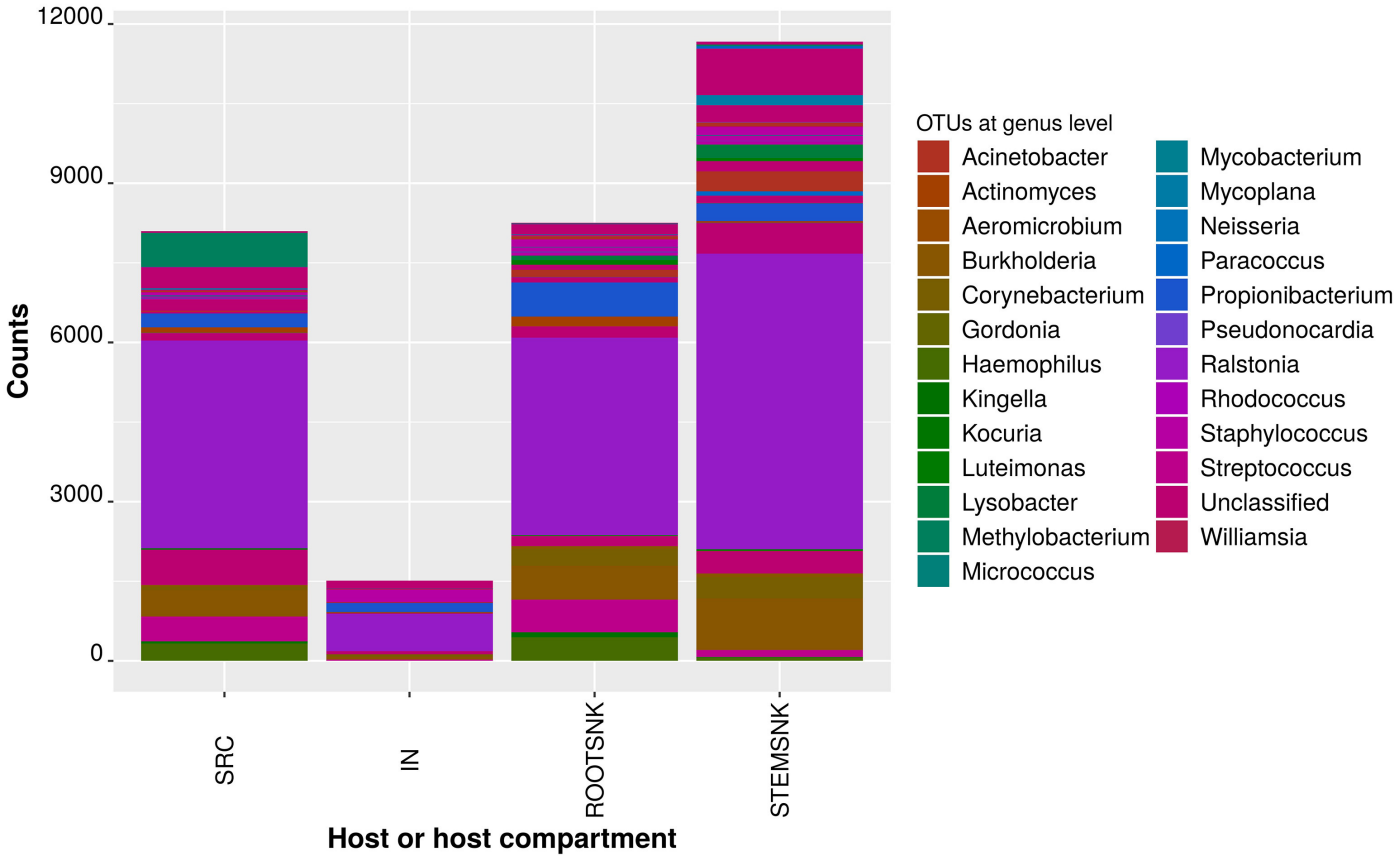
**Comparisons of statistically significant abundances per sample category in a rarified OTU table**. Frequencies per sample categories with non-normal distribution were compared using the Kruskal–Wallis test as implemented in QIIME. Only SRC, IN, STEMSNK, and ROOTSNK were analyzed. The probability of finding at least one mean significantly differing from the others was corrected using the False Discovery Rate (FDR) and the Bonferroni procedures, as implemented in QIIME.

Differentially abundant OTUs were analyzed for each host (Figure 4). Overall, 25 taxa were differentially abundant. A strong influence of the plant host was observed, since abundance of OTUs assigned to *H. parainfluenzae, Kingella* sp., *Brukholderia* sp., *Kocuria palustris, Acinetobacter* sp., *Neisseria* sp., *Micrococcus luteus*, and *Methyobacterium adhesivum* plummeted when passing from SRC to IN. We also observed how the sample type affected the relative abundances of specific OTUs.

In SRC, IN, STEMSNK, ROOTSNK and INSURF, the most abundant OTU was assigned to *Ralstonia* sp., with the highest number of sequences in the STEMSNK. In CTRLIN, CTRLROOT, and CTRLSTEM, the most abundant OTU was assigned to *Mycobacterium* sp., with the highest number of sequences in the CTRLSTEM (Supplementary Table 2).

**Figure 5:**
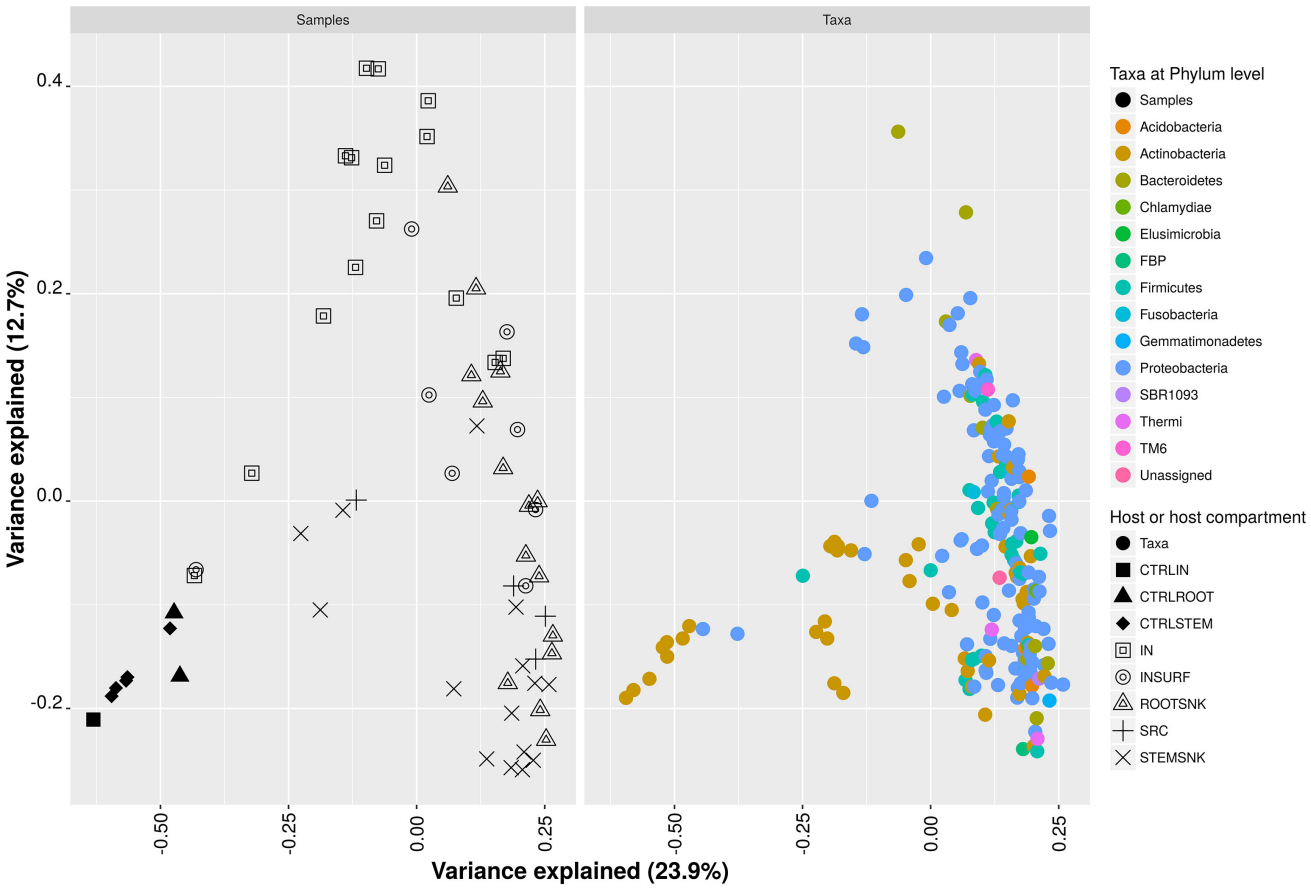
**Principal coordinate analysis of a rarefied OTU table at phylum level**. Dissimilarities were calculated using the Bray-Curtis distance estimator. Variance per axis is presented as percentage.

In contrast, in SRC, IN, STEMSNK, and INSURF, the least abundant OTU was *Paracoccus* sp. in ROOTSNK the least abundant OTU was assigned to *Staphylococcus* sp. in CTRLIN, CTRLROOT and CTRLSTEM, the least abundant OTU was also *Paracoccus* sp. comparison between samples indicated that bacterial communities are shaped by the insects or the plant and adapt to the hosts, altering the relative frequencies of key taxa (Figure 5). The analysis of principal coordinates showed that samples from SRC and ROOTSNK/STEMSNK grouped together, while control samples (CTRLROOT and CTRLSTEM as well as CTRLIN) formed a separate group. The IN samples separated from the rest of the groups. The microbiota of SRC and SNK showed that Proteobacteria, Actinobacteria, and Bacteroidetes grouped together with few *Chlamydia* sequences and some members of the TM6 clade of amoebal symbionts. In the CTRLIN, CTRLROOT, and CTRLSTEM, the Actinobacteria formed a separate group in the plot, suggesting differentiation from the rest of the community. The community in IN, predominantly comprising Proteobacteria, grouped together with Fusobacteria. The surface microbiota of IN appeared separate from IN and the plant samples (ROOTNSK and SRC) and was dominated by Proteobacteria. We further analyzed the dynamics of bacterial abundance fluctuation using ternary composition plots with bacterial endophytic communities of SRC, IN, STEMSNK, and ROOTSNK. This analysis suggested that some taxa were overgrown by others, in a host-dependent manner (Supplementary Figure 2). In the diagram, intersection of the perpendicular segments showed several phyla simultaneously. For example, at the tip of the triangle, a high density of OTUs was depicted. These OTUs were represented in lower abundances in SRC and IN (between 1 and 20%), while the same OTUs were in high abundance (80–100%) in the STEMSNK. Since diameter of the circles represents the relative abundance of OTUs in the three samples analyzed, the plot suggested that Actinobacteria were the most commonly found taxa in all samples (biggest circle is at coordinates 20% SRC, 1% IN, and 80% STEMSNK). In addition, a gradient could be seen where the community dominated by Proteobacteria and Actinobacteria in SRC and IN gradually changed to be composed of other groups like Firmucutes, Fusobacteria, and Acidobacteria. This gradient was easier to spot at the top vertex of the triangle, where a high density of different OTUs was seen at 100% abundance in the STEMSNK, as compared for example to the lower left vertex, where the community was mostly represented by Actinobacteria and Proteobacteria. In this gradient, emergence of Fusobacteria, Firmicutes and Bacteroidetes (although not highly represented in each sample) delineated a possible displacement of the initial-community dominated by Proteobacteria in SRC to a more diverse community in STEMSNK. Similar results were obtained for ROOTSNK, although Fusobacteria and Firmicutes had already been found in the IN sample (Supplementary Figure 2, lower right vertex of the triangle).

### Endophytes are acquired by insects through feeding and delivered to the stems of grapevine plants

**Figure 6:**
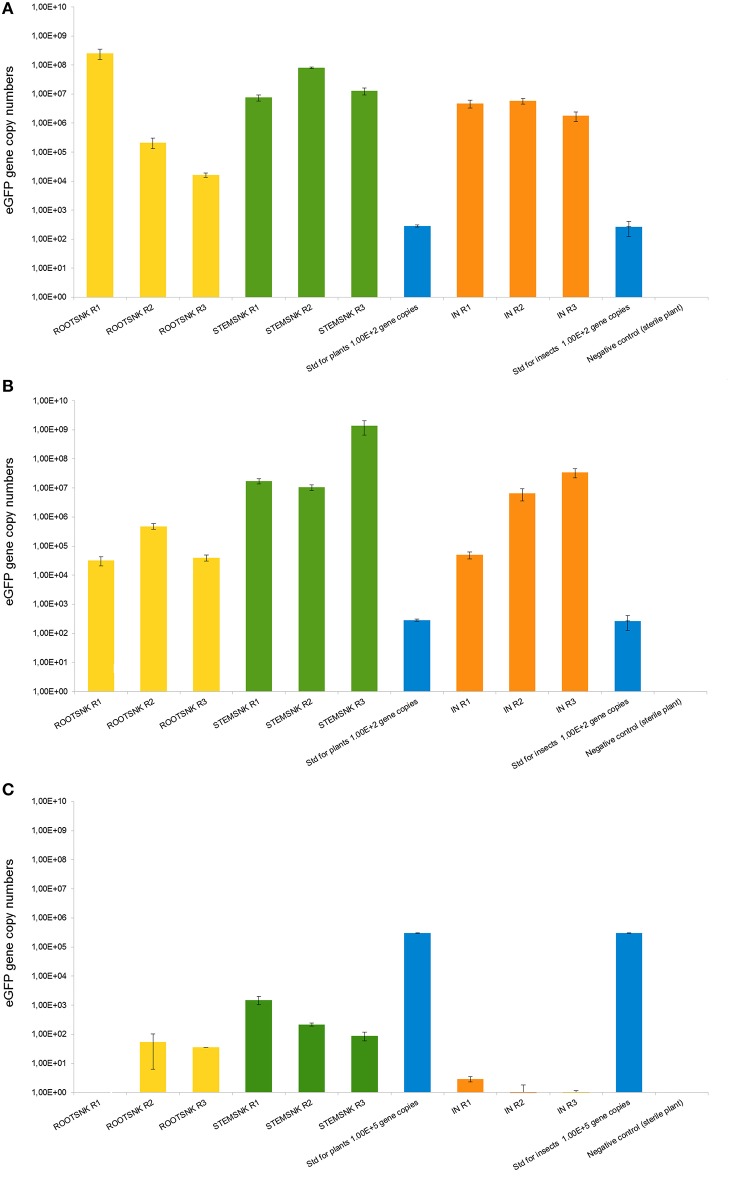
**Quantification of eGFP gene copy numbers in the plants and insects used in transmission experiments**. Gene copy numbers in the eGFP-bearing plasmid pMP4655 were quantified using qPCR with the second derivate method. For plants, the values obtained were benchmarked against the fresh weight (g) of samples; for insects, the copy numbers per individual are shown. Bars represent the mean of five biological replicates per treatment. Error bars represent the standard deviation of the mean. **(A)** Quantification of eGFP in *Enterobacter ludwigii* EnVs2. **(B)** Quantification of eGFP in *Enterobacter ludwigii* EnVs6. **(C)** Quantification of eGFP in *Pantoea vagans* PaVv9.

The qPCR analysis detected eGFP-tagged bacteria in both STEMSNK and ROOTSNK as well as in the IN vectors, confirming direct transmission of endophytes from insect to plant through feeding (Figure 6). In samples inoculated with *E. ludwigii* EnVs6 (pMP4655), we consistently detected more than 10^4^ eGFP gene copies/g of plant tissue in ROOTSNK, STEMSNK, and IN. eGFP quantification was lower (between 10^4^ and 10^6^ gene copies/g of plant tissue) in samples inoculated with *E. ludwigii* EnVs2 in ROOTSNK. However, the bacterium reached titers between 10^7^ and 10^9^ eGFP copies/insect in the STEMSNK samples and up to 10^7^ in IN samples.

In contrast, endophytic *P. vagans* PaVv9 was only partially transmitted. Quantification of gene copy numbers in SNK showed lower colonization levels of this endophyte, reaching only 10^2^ eGFP gene copies per gram of plant tissue in one STEMSNK, and being undetectable in one replicate of the ROOTSNK. The standard of 10^5^ gene copies per gram of plant tissue suggests complete amplification, confirming that the low numbers were the result of a poor transmission by the insect and not an artifact of the PCR.

We also performed endpoint PCR amplification in all the samples with specific primers for 16SrDNA genes of endosymbionts of *S. titanus*, i.e., *Cardinium* sp. and *Asaia* sp. We did detect *Asaia* sp. in the IN samples, though *Cardinium* sp. was not detected in either SRC, IN, or SNK.

## Discussion

Our experimental setup allowed us to confirm that *S. titanus* can transmit a wide variety of bacterial endophytes. This conclusion was based on common species content and diversity in the plants that were the source of inoculum (SRC), insects (IN) that serve as vectors and plants that were not hosting microorganisms and functioned as sinks (SNK), when IN fed on them (Figure 1).

When bacterial sequences were detected in only one of the hosts, i.e., SRC, IN, and SNK, we hypothesize that they were part of host microbiota, which was not transferred. On the contrary, when sequences were present in SRC, IN, and SNK, we hypothesize that the bacterial communities had been efficiently transferred from SRC to SNK through IN, in a contact-dependent manner. We also controlled for species transmitted from IN's own microbiomes by using the CTRLIN that had no contact with SRC and by sequencing the INSURF, detecting the community adhered to the insect's surface. When sequences were detected in the controls (CTRLSNK and CTRLIN) as well as in SRC, SNK, and IN, we speculate that these species belonged to the insect's microbiota and could be found in any of the samples that had contact with the insect.

In this context, we have shown that a vast part of the grapevine's endophytic bacterial community was efficiently vectored by *S. titanus* under laboratory conditions (Figure 2). We observed that insects selectively vectored endophytes and that bacterial communities were shaped by the vector insect (Figure 3 and Supplementary Table 1).

The communities we detected in SRC, IN, and SNK are distinctly characteristic of the grapevine's microbiota (Zarraonaindia et al., 2015). For example, the dominance of Proteobacteria has already been observed in *V. vinifera* cv. Tempranillo (Pinto et al., 2014), as well as the presence of Actinobacteria and Firmicutes (Zarraonaindia and Gilbert, 2015). The majority of genera encountered in our analysis have been reported in grapevine cultivars under different crop management strategies (Campisano et al., 2014a). Examples of those genera are *Flavobacterium, Staphylococcus*, and *Anaerococcus*. These genera, found consistently in independent experiments, constitute the core microbiome of grapevine (Pinto et al., 2014) and, not surprisingly, they were highly abundant in our samples. In addition, we detected sequences of *Propionibacterium acnes*, which has already been proposed to have co-evolved with grapevine (Campisano et al., 2014b). These findings open a new discussion on the origin of the core microbiota of grapevine and whether the insect's own symbionts can also be transferred and eventually acquired by grapevine plants, becoming part of that core microbiota. Two of *S. titanus* most important endosymbionts, namely *Cardinium* sp. (Sacchi et al., 2008) and *Asaia* sp., have been previously studied. These investigations have shown that *S. titanus* is able to deliver its *Cardinium* symbionts to artificial media amended with grapevine leaves, suggesting that even bacteria closely associated to IN could be transferred to the plant (Gonella et al., 2015). However, after searching for sequences of those two organisms within the pyrosequencing reads of all samples, we found no corresponding matches (data not shown). In addition, when performing endpoint PCR in SNK and IN samples, we did detect *Asaia* sp. in IN but not in samples of SNK. We also did not find *Cardinium* sp. in any of the samples, showing that endosymbionts are not evenly distributed in insect populations and that, in our case, most likely they were not transferred from IN to SNK.

Other transferred groups might have functional roles linked to colonization of SNK, since they could possess strategies that might be essential for correct establishment of the community, e.g., for cell-to-cell communication, as in *Agrobacterium* sp. and *Erwinia* sp. (Farrand et al., 2002; Hanano et al., 2014), siderophore and antimicrobial compound synthesis, as in *Stenotrophomonas* sp. (Ryan et al., 2009), or nitrogen metabolism (including nitrogen fixation).

We detected an overall fluctuation of diversity and species richness across samples. In Figure 3 and Supplementary Table 1, we show that richness drops following transfer from plant to vector, plausibly as an effect exerted by the host. It is well-known that microbial communities are adapted to very particular micro-environments in the host and this is one of the factors that shape microbiota structure. This effect can be seen when comparing microbiotas of non-phylogenetically related hosts, but also in closely related hosts, where the microbiota has a particular community structure. Studies have shown through pyrosequencing that different pig breeds harbor differential microbiota structures and that these can be transferred to non-related animals, e.g., mice, which in turn will also modify community structure, highlighting the specificity across hosts (Diao et al., 2016).

With these results, we have clearly outlined a pattern that shows that diversity is host-dependent. We describe this pattern in terms of ecological measurements. Greater variance in richness estimators (*Chao1* and observed species) denotes variable content of species per sample (Figure 3). In diversity estimators (Shannon-Wiener and Simpson), greater variance represents the non-dominance (equal distribution) of species, while small variances per sample represent dominance (unequal distribution) of a few species in the community. These effects can be easily seen in terms of Simpson's diversity estimator, where SRC's microbiota had a higher variance, which decreased as it was acquired by IN. In STEMSNK and ROOTSNK, variance of the estimator increased, once again suggesting the non-dominance of particular species. When analyzing these fluctuations as a composite, we are observing an “expansion and contraction” phenomenon of the bacterial endophytic community where changing hosts alters the community structure and possibly changes in functional groups. From this perspective, species dominance might hint at a bottleneck for specialists (bacteria with restricted enzymatic capabilities) that might be increased and dominate when inhabiting the IN. When inhabiting the plant hosts (SRC and SNK), generalists (e.g., bacteria with a wider range of enzymes capable of using resources in the ecosystem) might be more evenly distributed.

An explanation of such generalists in the SRC might be their origin. These plants originated from a commercial nursery and were grown in a greenhouse. Therefore, they are a rich source of inoculum. Most probably, they have acquired their endophytic bacterial communities from the soil (Vandenkoornhuyse et al., 2015), which is a reservoir for generalists (Monard et al., 2016). However, changing from plant to animal host might mean a challenge for the generalists that are unable to produce adequate enzymes for nutrition with the insect's resources resulting in a decrease in abundance. Nutrients in the insect host differ from those in the plant, which may also affect the way the community copes during adaptation. As an example, matrices in each host differ in composition. In plants, phloem is a complex matrix. Seventy percent of the carbohydrate content in the phloem of *V. vinifera* is comprised of sucrose, with lower quantities of glucose and fructose. The main amino acids found are glutamine and proline as well as organic substances such as tartaric, malic, and citric acids (Glad et al., 1992). In contrast, large molecules like sialic acid, collagen, and chitin are present in the animal tissues, limiting growth of some bacteria (Thomas, 2016). Other factors affecting community structure when switching hosts might be genetic mutations in single alleles of bacteria (Viana et al., 2015) as well as “a criterion for proximity,” where symbionts spatially distant from target hosts are less capable of changing hosts, thus adapting to this selective pressure (van Baarlen et al., 2007).

Not only nutritional factors may be involved in altering community structure. Niche occupation might be a trigger for competition among bacteria. In our experiments, niches in SRC's microbiota may have overlapped with those of IN's microbiota resulting in competitive exclusion. This could result in a decrease in the number of species from SRC to IN when abundance of some genera drastically changed from plant to insect (Figure 4 and Supplementary Table 2). Among those microorganisms whose abundance decreased, we found plant- and human-associated bacteria that have been previously identified in the microbiome of grapevine (Yousaf et al., 2014). We believe that these bacteria might behave as drivers of diversity with roles in directing community structure during host colonization, given the specificity of their change in abundance and because they harbor genetic determinants that make them good competitors. Other such drivers are present in two cases: (i) those whose abundance increased after being transferred from IN to SNK and (ii) those whose abundance increased from STEMSNK to ROOTSNK. In the first case (IN to SNK), *Ralstonia, Propionibacterium, Burkholderia*, and *Staphylococcus* were the genera whose abundance most drastically changed. These organisms have different functions related to colonization pioneering. For example, the genera *Ralstonia* and *Burkholderia* have a great adaptability for host selection and a wide range of mechanisms for colonization (Compant et al., 2005; Genin, 2010). Both genera include plant pathogens as well as symbionts, reflecting genome plasticity. *Propionibacterium* and *Staphylococcus* are symbionts that have co-evolved with humans and are rich in enzymatic activities. These enzymes could be involved in the selection of other microbes that become part of the endophytic community to be transferred or in the conditioning of the micro-habitat before colonization by other microbes.

In the second case (transfer from STEMSNK to ROOTSNK), *Haemophilus, Paracoccus, Rhodococcus*, and *Micrococcus* were differentially represented in the community of stems and roots. A possible explanation of this shift may have a nutritional basis. Root exudates may have a strong influence on the assemblage of microbial communities in the rhizosphere (Shi et al., 2011). For example, root exudates of rice plants grown under hydroponic conditions contain large amounts of carbohydrates and amino acids that play a role as chemoattractants for endophytic bacteria (Bacilio-Jimenez et al., 2003). Rhizodeposition, the process of exudate production and microenvironment enrichment driven by root cap cell deposition, is important in luring bacteria to the endosphere (Bulgarelli et al., 2013). In grapevine, the effect of root exudates on colonization by endophytes is not well-established, however we speculate that root exudates do influence root microbiota, helping differentiate the communities in the STEMSNK and ROOTSNK. Root exudates can diffuse out to the rhizosphere and into the plant and thus have an effect on patterns of microbial colonization of the root, even from the inside.

As opposed to the SRC, SNK and IN, CTRLSTEM, CTRLROOT, and CTRLIN clearly differed in terms of community composition, where Actinobacteria and Proteobacteria were completely dominant (Figures 1, 3). Only few of the transferred endophytes found in SRC, SNK, and IN were found in the controls.

We have also shown a distinction between the insect's microbiota and the plant's endophytic community and propose differentiation according to the host (Figure 5). Shifts in the bacterial community are associated with a gradient where some of the taxonomical groups have more weight during transmission than the others (Supplementary Figures 2, 3). Such a differentiation suggests that some taxa are more proliferous in particular hosts. This is of extreme importance in terms of possible endophytic microbiota manipulation by engineering. If some of the endophytes (for example those assigned to Bacteroidetes in IN or Acidobacteria in SRC) can grow better in certain types of hosts, we might drive our efforts to enrich and improve these groups in those hosts and experiment to further analyze their plant protection properties for use in agriculture. With our experiments, we can also predict that those groups that acquire greater importance in particular hosts might also be safe to apply in crops of economic value, since they behave as native microbiota.

We also highlight the selectivity of the insect as a vector of endophytes. Our qPCR experiments (Figure 6) suggest that endophytes like *E. ludwigii* EnVs2 and *Enterobacter* sp. LecVs2 are effectively vectored by the insect from an exogenous source. However, for endophyte *P. vagans* PaVv9, the transmission was inefficient. It is possible that only few members of the endophytic community that are acquired by the insect will survive in this host. This kind of selectivity has been observed in pathogens of grapevine. For example, *S. titanus* is able to transfer the FDP more efficiently than any other leafhopper vector (Chuche and Thiéry, 2014). This, together with the high grapevine specificity could make *S. titanus* an ideal candidate for endophytic delivery in future therapeutic applications in the vineyards.

Being isolated from healthy plants, we speculate that the transferred symbionts have a mutualistic association with grapevine. Previous studies showed that grapevine endophytes benefit the plant by stimulating growth and protecting it from incoming pathogens (Campisano et al., 2015). In addition, genomic studies of selected endophytic strains from grapevine revealed their potential as plant protection agents (Lòpez-Fernàndez et al., 2015). In those studies, strains *E. ludwigii* EnVs6, *P. vagans* PaVv9, and *E. ludwigii* LecVs2 acted as good plant growth promoters and biocontrol agents and had interesting genomic traits for bioprospecting. In the present study these strains were used to prove that they are transferred by *S. titanus* (Figure 6). Results from these experiments suggest that members that are actively transmitted by the insect can possess beneficial properties for the plant. Although it may not be the case for all members in the transferred endophytic community, we believe that the endomicrobiome is populated with mutualists that provide the plant with fitness and ecological advantages. Moreover, as we have seen in our experiments, beneficial endophytes might be selectively transferred by the insect, hinting at the possibility of insect mediated community shaping.

Studying the transmission of the bacterial endophytic communities in economically relevant crops such as grapevine could revolutionize the approaches through which we have dealt with plant disease in the past. In addition, by depicting the structure of the bacterial communities of endophytes that can be transferred, it is possible to identify recalcitrant microbes, drivers of diversity and markers that should be further studied from a metabolic point of view. Vectoring of beneficial bacteria is an important issue for agricultural research, and possibly comparable to the transmission of pathogens in other systems, e.g., as in humans. However, to our knowledge, few works have tried to disentangle the events that occur during the transmission of such beneficial microbes. Moreover, the amount of experimental evidence supporting the enrichment of beneficial microbes in agricultural systems, where they actually have an impact on a larger scale is negligible. With our work, we have provided more evidence for the vector-assisted transmission of endophytes and propose further investigation where microbiota engineering and wider transmission experiments should be done in order to understand if beneficial microbes could support or even replace the use of other methods for plant protection, including genetic modifications in plants or employment of chemical pesticides.

## Author contributions

AC, VM, and IP conceived the experiments and contributed to the bioinformatic analysis of pyrosequencing data. SL performed all the experiments, performed data analysis, and wrote the manuscript. VM contributed to the design and interpretation of experiments with insects, FP contributed to the design and performance of qPCR experiments and all the co-authors contributed to a critical revision of the document.

### Conflict of interest statement

The authors declare that the research was conducted in the absence of any commercial or financial relationships that could be construed as a potential conflict of interest.

## Supplementary material

Supplementary Figure 1 **Rarefaction curves per sample category using a sampling depth of 10–3,000 sequences per sample**. Bars represent the standard deviation of the mean.

Supplementary Figure 2 **Ternary plots based on a rarified OTU table at the phylum level**. Ternary plots were drawn using the software ggtern (Hamilton, 2016). Circles represent the mean of the total observations of a particular phylum in all samples. Each triangle side corresponds to a host and the colored grid corresponds to abundance percentages in each host, from 0 to 100%. The plot was depicted using STEMSNK as a host compartment for analysis.

Supplementary Figure 3 **Ternary plots based on a rarified OTU table at the phylum level**. Ternary plots were drawn using the software ggtern (Hamilton, 2016). Circles represent the mean of the total observations of a particular phylum in all samples. Each triangle side corresponds to a host and the colored grid corresponds to abundance percentages in each host, from 0 to 100%. The plot was depicted using ROOTSNK as a host compartment for analysis.

Supplementary Table 1 **(A)** Diversity indexes calculated for the endophytic community transmitted across grapevine by *S. titanus*. **(B)** Richness and diversity analysis using paired non-parametric *t*-test (Montecarlo permutations) with α = 0.05.

Supplementary Table 2 **Differentially abundant taxa in a Kruskal–Wallis test with α = 0.05**.

## References

[B1] Atangana A. Khasa D. Chang S. Degrande A. (2014). Ecological interactions and productivity in agroforestry systems, in Tropical Agroforestry (Dordrecht: Springer Netherlands), 151–172. Available online at: http://link.springer.com/10.1007/978-94-007-7723-1_7 (Accessed January 17, 2017).

[B2] Bacilio-Jimenez M. Aguilar-Flores S. Ventura-Zapata E. Pérez-Campos E. Bouquelet S. (2003). Chemical characterization of root exudates from rice (Oryza sativa) and their effects on the chemotactic response of endophytic bacteria. Plant Soil 2, 271–277. 10.1023/A:1022888900465

[B3] Bloemberg G. V. Wijfjes A. H. M. Lamers G. E. M. Stuurman N. Lugtenberg B. J. J. (2000). Simultaneous imaging of *Pseudomonas fluorescens* WCS365 populations expressing three different autofluorescent proteins in the rhizosphere: new perspectives for studying microbial communities. Mol. Plant Microbe Interact. 13, 1170–1176. 10.1094/MPMI.2000.13.11.1170 11059483

[B4] Bright M. Bulgheresi S. (2010). A complex journey: transmission of microbial symbionts. Nat. Rev. Microbiol. 8, 218–230. 10.1038/nrmicro2262 20157340 PMC2967712

[B5] Bulgarelli D. Schlaeppi K. Spaepen S. van Themaat E. V. L. Schulze-Lefert P. (2013). Structure and functions of the bacterial microbiota of plants. Annu. Rev. Plant Biol. 64, 807–838. 10.1146/annurev-arplant-050312-120106 23373698

[B6] Campisano A. Antonielli L. Pancher M. Yousaf S. Pindo M. Pertot I. (2014a). Bacterial endophytic communities in the grapevine depend on pest management. PLoS ONE 9:e112763. 10.1371/journal.pone.0112763 25387008 PMC4227848

[B7] Campisano A. Ometto L. Compant S. Pancher M. Antonielli L. Yousaf S.. (2014b). Interkingdom transfer of the acne-causing agent, *Propionibacterium acnes*, from human to grapevine. Mol. Biol. Evol. 31, 1059–1065. 10.1093/molbev/msu075 24554779

[B8] Campisano A. Pancher M. Puopolo G. Puddu A. Lòpez-Fernàndez S. Biagini B. (2015). Diversity in endophyte populations reveals functional and taxonomic diversity between wild and domesticated grapevines. Am. J. Enol. Vitic. 66, 12–21. 10.5344/ajev.2014.14046

[B9] Caporaso J. G. Bittinger K. Bushman F. D. DeSantis T. Z. Andersen G. L. Knight R. (2010a). PyNAST: a flexible tool for aligning sequences to a template alignment. Bioinformatics 26, 266–267. 10.1093/bioinformatics/btp636 19914921 PMC2804299

[B10] Caporaso J. G. Kuczynski J. Stombaugh J. Bittinger K. Bushman F. D. Costello E. K.. (2010b). QIIME allows analysis of high-throughput community sequencing data. Nat. Methods 7, 335–336. 10.1038/nmeth.f.303 20383131 PMC3156573

[B11] Chuche J. Thiéry D. (2014). Biology and ecology of the Flavescence dorée vector *Scaphoideus titanus*: a review. Agron. Sustain. Dev. 34, 381–403. 10.1007/s13593-014-0208-7

[B12] Chuche J. Thiéry D. Mazzoni V. (2011). Do *Scaphoideus titanus* (Hemiptera: Cicadellidae) nymphs use vibrational communication? Naturwissenschaften 98, 639–642. 10.1007/s00114-011-0808-x 21656005

[B13] Compant S. Clément C. Sessitsch A. (2010). Plant growth-promoting bacteria in the rhizo- and endosphere of plants: their role, colonization, mechanisms involved and prospects for utilization. Soil Biol. Biochem. 42, 669–678. 10.1016/j.soilbio.2009.11.024

[B14] Compant S. Kaplan H. Sessitsch A. Nowak J. Ait Barka E. Clèment C. (2008). Endophytic colonization of *Vitis vinifera* L. by *Burkholderia phytofirmans* strain PsJN: from the rhizosphere to inflorescence tissues: FEMS Microbiol. Ecol. 63, 84–93. 10.1111/j.1574-6941.2007.00410.x 18081592

[B15] Compant S. Muzammil S. Lebrihi A. Mathieu F. (2013). Visualization of grapevine root colonization by the Saharan soil isolate *Saccharothrix algeriensis* NRRL B-24137 using DOPE-FISH microscopy. Plant Soil 370, 583–591. 10.1007/s11104-013-1648-6

[B16] Compant S. Reiter B. Sessitsch A. Nowak J. Clement C. Ait Barka E. (2005). Endophytic Colonization of *Vitis vinifera* L. by plant growth-promoting bacterium *Burkholderia* sp. strain PsJN. Appl. Environ. Microbiol. 71, 1685–1693. 10.1128/AEM.71.4.1685-1693.2005 15811990 PMC1082517

[B17] Coveri P. (1992, April 12). Associazione Costitutori Vitticoi Italiani (ACOVIT). Varietà Pinot Nero N. Available online at: https://www.acovit.it/uve-da-vino/149-pinot-nero-n.

[B18] DeSantis T. Z. Hugenholtz P. Larsen N. Rojas M. Brodie E. L. Keller K.. (2006). Greengenes, a chimera-checked 16S rRNA gene database and workbench compatible with ARB. Appl. Environ. Microbiol. 72, 5069–5072. 10.1128/AEM.03006-05 16820507 PMC1489311

[B19] Diao H. Yan H. L. Xiao Y. Yu B. Yu J. He J.. (2016). Intestinal microbiota could transfer host Gut characteristics from pigs to mice. BMC Microbiol. 16:238. 10.1186/s12866-016-0851-z 27729007 PMC5057279

[B20] Edgar R. C. (2010). Search and clustering orders of magnitude faster than BLAST. Bioinformatics 26, 2460–2461. 10.1093/bioinformatics/btq461 20709691

[B21] Farrand S. K. Qin Y. Oger P. (2002). Quorum-sensing system of *Agrobacterium* plasmids: analysis and utility, in Methods in Enzymology (Elsevier), 452–484. Available online at: http://linkinghub.elsevier.com/retrieve/pii/S0076687902581088 (Accessed January 17, 2017). 10.1016/s0076-6879(02)58108-8 12474406

[B22] Foissac X. Wilson M. R. (2009). Current and possible future distributions of phytoplasma diseases and their vectors, in Phytoplasmas: Genomes, Plant Hosts and Vectors, eds Weintraub P. G. Jones P. (Wallingford, CT: CABI), 309–324. Available online at: http://www.cabi.org/cabebooks/ebook/20093353140 (Accessed January 17, 2017).

[B23] Gai C. S. Lacava P. T. Quecine M. C. Auriac M.-C. Lopes J. R. S. Araújo W. L.. (2009). Transmission of *Methylobacterium mesophilicum* by *Bucephalogonia xanthophis* for paratransgenic control strategy of Citrus variegated chlorosis. J. Microbiol. 47, 448–454. 10.1007/s12275-008-0303-z 19763419

[B24] Galetto L. Miliordos D. Pegoraro M. Sacco D. Veratti F. Marzachì C.. (2016). Acquisition of Flavescence Dorée phytoplasma by *Scaphoideus titanus* Ball from different grapevine varieties. Int. J. Mol. Sci. 17:1563. 10.3390/ijms17091563 27649162 PMC5037832

[B25] Genin S. (2010). Molecular traits controlling host range and adaptation to plants in *Ralstonia solanacearum*: research review. New Phytol. 187, 920–928. 10.1111/j.1469-8137.2010.03397.x 20673287

[B26] Glad C. Regnard J. L. Querou Y. Brun O. Morot-Gaudry J. F. (1992). Phloem sap exudates as a criterion for sink strength appreciation in *Vitis vinifera* cv. Pinot noir grapevines. Vitis 31, 131–138.

[B27] Gonella E. Pajoro M. Marzorati M. Crotti E. Mandrioli M. Pontini M.. (2015). Plant-mediated interspecific horizontal transmission of an intracellular symbiont in insects. Sci. Rep. 5:15811. 10.1038/srep15811 26563507 PMC4643326

[B28] Hamilton N. (2016). ggtern: An Extension to ‘ggplot2’, for the Creation of Ternary Diagrams. R Package Version 2.1.5. Available online at: https://CRAN.R-project.org/package=ggtern

[B29] Hanahan D. (1983). Studies on transformation of *Escherichia coli* with plasmids. J. Mol. Biol. 166, 557–580. 10.1016/S0022-2836(83)80284-8 6345791

[B30] Hanano A. Harba M. Al-Ali M. Ammouneh H. (2014). Silencing of *Erwinia amylovora* sy69 AHL-quorum sensing by a *Bacillus simplex* AHL-inducible *aiiA* gene encoding a zinc-dependent *N*- acyl-homoserine lactonase. Plant Pathol. 63, 773–783. 10.1111/ppa.12142

[B31] Harris K. F. Maramorosch K. (eds.) (1980). Vectors of Plant Pathogens. New York, NY: Academic Press.

[B32] Iniguez A. L. Dong Y. Carter H. D. Ahmer B. M. M. Stone J. M. Triplett E. W. (2005). Regulation of enteric endophytic bacterial colonization by plant defenses. Mol. Plant. Microbe Interact. 18, 169–178. 10.1094/MPMI-18-0169 15720086

[B33] Jermini M. Morisoli R. Rigamoni I. Girgenti P. Mazzoni V. (2015). Fertility, longevity, oviposition dynamic and sex-ratio of *Scaphoideus titanus* Ball, in IOBC-WPRS Meeting of the Working Group on “Integrated Protection and Production in Viticulture”, (Vienna). (Accessed October 20–23, 2015).

[B34] Khan A. Hamayun M. Kang S.-M. Kim Y.-H. Jung H.-Y. Lee J.-H.. (2012). Endophytic fungal association via gibberellins and indole acetic acid can improve plant growth under abiotic stress: an example of *Paecilomyces formosus* LHL10. BMC Microbiol. 12:3. 10.1186/1471-2180-12-3 22235902 PMC3268082

[B35] Kusari P. Kusari S. Spiteller M. Kayser O. (2015). Implications of endophyte-plant crosstalk in light of quorum responses for plant biotechnology. Appl. Microbiol. Biotechnol. 99, 5383–5390. 10.1007/s00253-015-6660-8 25971199

[B36] Lòpez-Fernàndez S. Sonego P. Moretto M. Pancher M. Engelen K. Pertot I.. (2015). Whole-genome comparative analysis of virulence genes unveils similarities and differences between endophytes and other symbiotic bacteria. Front. Microbiol. 6:419. 10.3389/fmicb.2015.00419 26074885 PMC4443252

[B37] Médiène S. Valantin-Morison M. Sarthou J.-P. de Tourdonnet S. Gosme M. Bertrand M. (2011). Agroecosystem management and biotic interactions: a review. Agron. Sustain. Dev. 31, 491–514. 10.1007/s13593-011-0009-1

[B38] Mercado-Blanco J. Lugtenberg B. (2014). Biotechnological applications of bacterial endophytes. Curr. Biotechnol. 3, 60–75. 10.2174/22115501113026660038

[B39] Møller T. S. B. Overgaard M. Nielsen S. S. Bortolaia V. Sommer M. O. A. Guardabassi L.. (2016). Relation between tetR and tetA expression in tetracycline resistant *Escherichia coli*. BMC Microbiol. 16:39. 10.1186/s12866-016-0649-z 26969122 PMC4788846

[B40] Monard C. Gantner S. Bertilsson S. Hallin S. Stenlid J. (2016). Habitat generalists and specialists in microbial communities across a terrestrial-freshwater gradient. Sci. Rep. 6:37719. 10.1038/srep37719 27886241 PMC5123577

[B41] Pancher M. Ceol M. Corneo P. E. Longa C. M. O. Yousaf S. Pertot I.. (2012). Fungal endophytic communities in grapevines (*Vitis vinifera* L.) respond to crop management. Appl. Environ. Microbiol. 78, 4308–4317. 10.1128/AEM.07655-11 22492448 PMC3370515

[B42] Pèrez-Brocal V. Latorre A. Moya A. (2013). Symbionts and pathogens: what is the difference? in Symbionts and pathogens: what is the difference? eds Dobrindt U. Hacker H. H. Svanborg C. (New York, NY; Heidelberg: Springer), 215–243. 10.1007/82_2011_190 22076025

[B43] Pinto C. Pinho D. Sousa S. Pinheiro M. Egas C. Gomes A. C. (2014). Unravelling the diversity of grapevine microbiome. PLoS ONE 9:e85622. 10.1371/journal.pone.0085622 24454903 PMC3894198

[B44] Quadt-Hallmann A. Kloepper J. W. Benhamou N. (1997). Bacterial endophytes in cotton: mechanisms of entering the plant. Can. J. Microbiol. 43, 577–582. 10.1139/m97-081

[B45] Rabha A. J. Naglot A. Sharma G. D. Gogoi H. K. Veer V. (2014). *In vitro* evaluation of antagonism of endophytic *Colletotrichum gloeosporioides* against potent fungal pathogens of *Camellia sinensis*. Indian J. Microbiol. 54, 302–309. 10.1007/s12088-014-0458-8 24891737 PMC4039731

[B46] Reinhold-Hurek B. Hurek T. (2011). Living inside plants: bacterial endophytes. Curr. Opin. Plant Biol. 14, 435–443. 10.1016/j.pbi.2011.04.004 21536480

[B47] Ryan R. P. Monchy S. Cardinale M. Taghavi S. Crossman L. Avison M. B.. (2009). The versatility and adaptation of bacteria from the genus *Stenotrophomonas*. Nat. Rev. Microbiol. 7, 514–525. 10.1038/nrmicro2163 19528958

[B48] Sacchi L. Genchi M. Clementi E. Bigliardi E. Avanzati A. M. Pajoro M.. (2008). Multiple symbiosis in the leafhopper *Scaphoideus titanus* (Hemiptera: Cicadellidae): details of transovarial transmission of *Cardinium* sp. and yeast-like endosymbionts. Tissue Cell 40, 231–242. 10.1016/j.tice.2007.12.005 18272191

[B49] Santoyo G. Moreno-Hagelsieb G. del Carmen Orozco-Mosqueda M. Glick B. R. (2016). Plant growth-promoting bacterial endophytes. Microbiol. Res. 183, 92–99. 10.1016/j.micres.2015.11.008 26805622

[B50] Schulz B. Boyle C. (2006). What are Endophytes? in Microbial Root Endophytes, eds Schulz B. J. E. Boyle C. J. C. Sieber T. N. (Berlin; Heidelberg: Springer), 1–13. Available online at: http://link.springer.com/10.1007/3-540-33526-9_1 (Accessed January 17, 2017).

[B51] Shi S. Richardson A. E. O'Callaghan M. DeAngelis K. M. Jones E. E. Stewart A.. (2011). Effects of selected root exudate components on soil bacterial communities. FEMS Microbiol. Ecol. 77, 600–610. 10.1111/j.1574-6941.2011.01150.x 21658090

[B52] Thomas G. H. (2016). Sialic acid acquisition in bacteria-one substrate, many transporters. Biochem. Soc. Trans. 44, 760–765. 10.1042/BST20160056 27284039

[B53] Truyens S. Weyens N. Cuypers A. Vangronsveld J. (2015). Bacterial seed endophytes: genera, vertical transmission and interaction with plants: bacterial seed endophytes. Environ. Microbiol. Rep. 7, 40–50. 10.1111/1758-2229.12181

[B54] van Baarlen P. van Belkum A. Summerbell R. C. Crous P. W. Thomma B. P. H. J. (2007). Molecular mechanisms of pathogenicity: how do pathogenic microorganisms develop cross-kingdom host jumps? FEMS Microbiol. Rev. 31, 239–277. 10.1111/j.1574-6976.2007.00065.x 17326816

[B55] Vandenkoornhuyse P. Quaiser A. Duhamel M. Le Van A. Dufresne A. (2015). The importance of the microbiome of the plant holobiont. New Phytol. 206, 1196–1206. 10.1111/nph.13312 25655016

[B56] Viana D. Comos M. McAdam P. R. Ward M. J. Selva L. Guinane C. M.. (2015). A single natural nucleotide mutation alters bacterial pathogen host tropism. Nat. Genet. 47, 361–366. 10.1038/ng.3219 25685890 PMC4824278

[B57] Weintraub P. G. Beanland L. (2006). Insect vectors of phytoplasmas. Annu. Rev. Entomol. 51, 91–111. 10.1146/annurev.ento.51.110104.151039 16332205

[B58] Yousaf S. Bulgari D. Bergna A. Pancher M. Quaglino F. Casati P.. (2014). Pyrosequencing detects human and animal pathogenic taxa in the grapevine endosphere. Front. Microbiol. 5:327. 10.3389/fmicb.2014.00327 25071740 PMC4085568

[B59] Zarraonaindia I. Gilbert J. (2015). Understanding grapevine-microbiome interactions: implications for viticulture industry. Microb. Cell 2, 171–173. 10.15698/mic2015.05.204 28357290 PMC5349240

[B60] Zarraonaindia I. Owens S. M. Weisenhorn P. West K. Hampton-Marcell J. Lax S.. (2015). The soil microbiome influences grapevine-associated microbiota. mBio 6:e02527–14. 10.1128/mBio.02527-14 25805735 PMC4453523

