# Supplementary figures and images for: A Phloem-Feeding Insect Transfers Bacterial Endophytic Communities between Grapevine Plants
Source: Front Microbiol. 2017 May 15;8:834. doi: 10.3389/fmicb.2017.00834 (PMC5430944; doi:10.3389/fmicb.2017.00834)

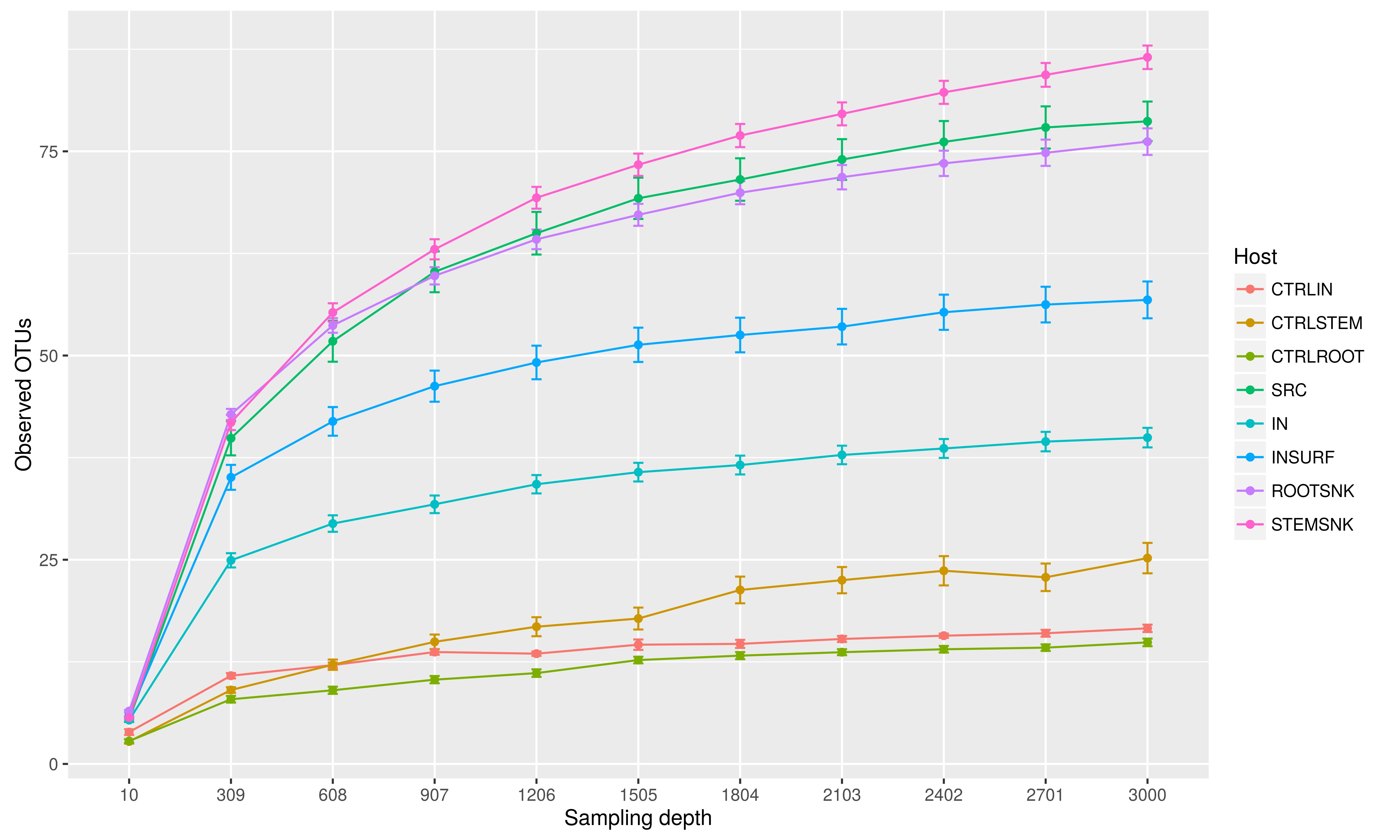

Supplement: Supplementary Figure 1 — Rarefaction curves per sample category using a sampling depth of 10–3,000 sequences per sample. Bars represent the standard deviation of the mean. [file Image1.JPEG]

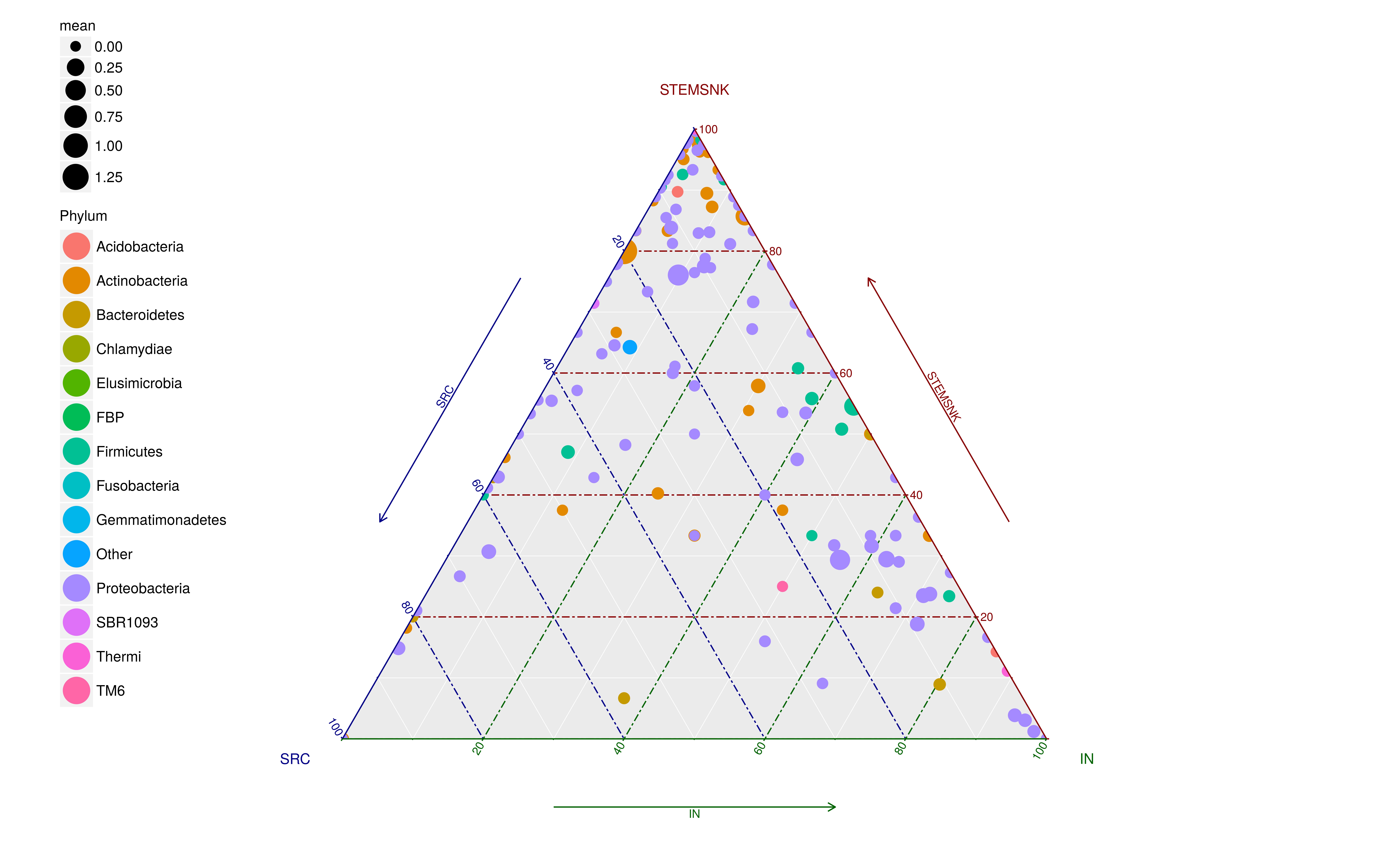

Supplement: Supplementary Figure 2 — Ternary plots based on a rarified OTU table at the phylum level. Ternary plots were drawn using the software ggtern (Hamilton, 2016). Circles represent the mean of the total observations of a particular phylum in all samples. Each triangle side corresponds to a host and the colored grid corresponds to abundance percentages in each host, from 0 to 100%. The plot was depicted using STEMSNK as a host compartment for analysis. [file Image2.JPEG]

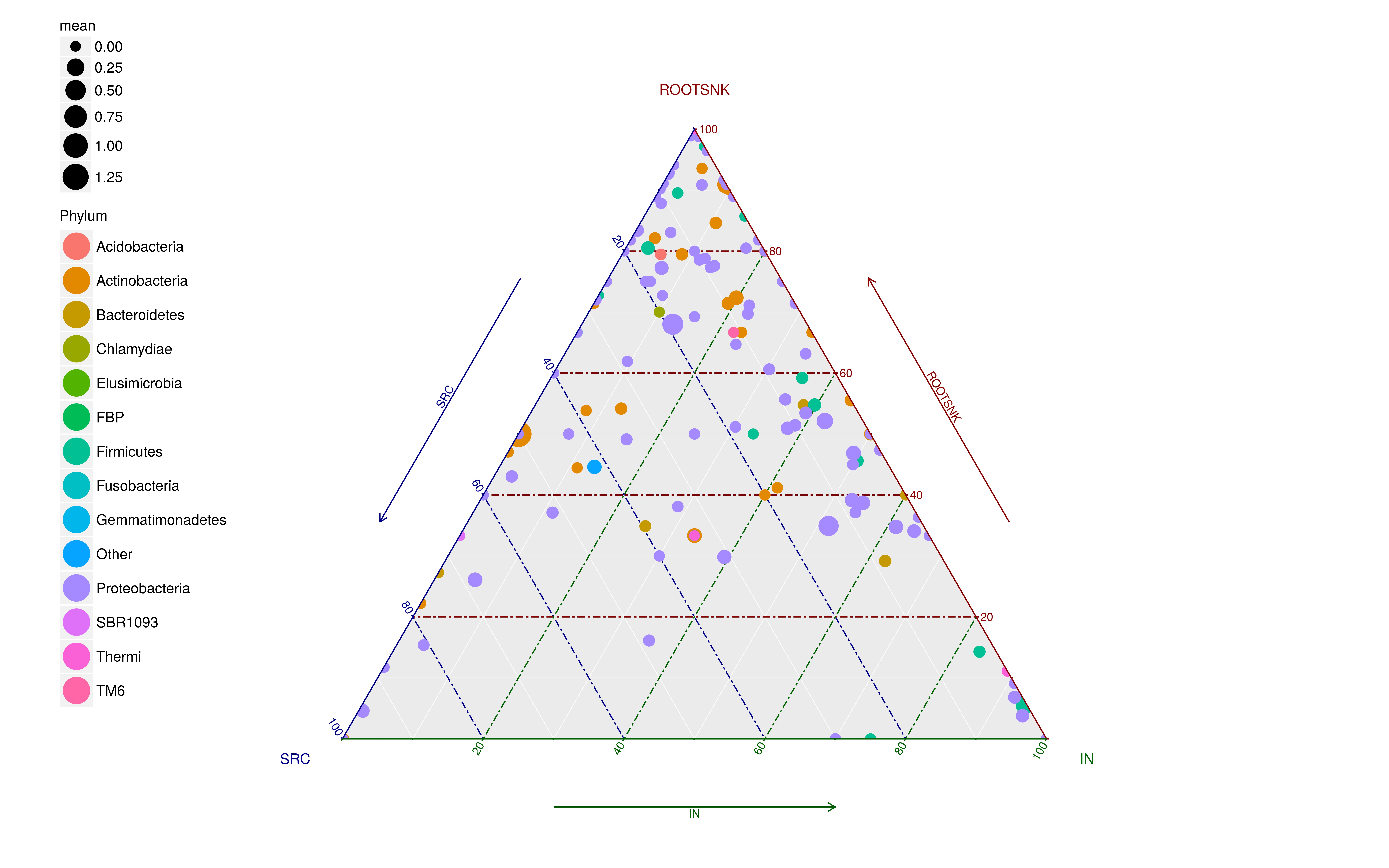

Supplement: Supplementary Figure 3 — Ternary plots based on a rarified OTU table at the phylum level. Ternary plots were drawn using the software ggtern (Hamilton, 2016). Circles represent the mean of the total observations of a particular phylum in all samples. Each triangle side corresponds to a host and the colored grid corresponds to abundance percentages in each host, from 0 to 100%. The plot was depicted using ROOTSNK as a host compartment for analysis. [file Image3.JPEG]
